# Silencing of miR-182 is associated with modulation of tumorigenesis through apoptosis induction in an experimental model of colorectal cancer

**DOI:** 10.1186/s12885-019-5982-9

**Published:** 2019-08-20

**Authors:** Lisa Perilli, Sofia Tessarollo, Laura Albertoni, Matteo Curtarello, Anna Pastò, Efrem Brunetti, Matteo Fassan, Massimo Rugge, Stefano Indraccolo, Alberto Amadori, Stefania Bortoluzzi, Paola Zanovello

**Affiliations:** 1 Immunology and Molecular Oncology Unit, Veneto Institute of Oncology IOV – IRCCS, Padua, Italy; 2 Genetics and Molecular Biology Unit, ULSS 8 Berica, Vicenza, Italy; 3 Surgical Pathology and Cytopathology Unit, Department of Medicine, University of Padova, Padua, Italy; 4 Department of Surgery, Oncology and Gastroenterology, Immunology & Oncology Section, University of Padova, Padua, Italy; 5 Department of Molecular Medicine, University of Padova, Padua, Italy

**Keywords:** Colorectal cancer, microRNA, Apoptosis, Cell proliferation, Tumorigenesis

## Abstract

**Background:**

miR-182-5p (miR-182) is an oncogenic microRNA (miRNA) found in different tumor types and one of the most up-regulated miRNA in colorectal cancer (CRC). Although this microRNA is expressed in the early steps of tumor development, its role in driving tumorigenesis is unclear.

**Methods:**

The effects of miR-182 silencing on transcriptomic profile were investigated using two CRC cell lines characterized by different in vivo biological behavior, the MICOL-14^h-tert^ cell line (dormant upon transfer into immunodeficient hosts) and its tumorigenic variant, MICOL-14^tum^. Apoptosis was studied by annexin/PI staining and cleaved Caspase-3/PARP analysis. The effect of miR-182 silencing on the tumorigenic potential was addressed in a xenogeneic model of MICOL-14^tum^ transplant.

**Results:**

Endogenous miR-182 expression was higher in MICOL-14^tum^ than in MICOL-14^h-tert^ cells. Interestingly, miR-182 silencing had a strong impact on gene expression profile, and the positive regulation of apoptotic process was one of the most affected pathways. Accordingly, annexin/PI staining and caspase-3/PARP activation demonstrated that miR-182 treatment significantly increased apoptosis, with a prominent effect in MICOL-14^tum^ cells. Moreover, a significant modulation of the cell cycle profile was exerted by anti-miR-182 treatment only in MICOL-14^tum^ cells, where a significant increase in the fraction of cells in G0/G1 phases was observed. Accordingly, a significant growth reduction and a less aggressive histological aspect were observed in tumor masses generated by in vivo transfer of anti-miR-182-treated MICOL-14^tum^ cells into immunodeficient hosts.

**Conclusions:**

Altogether, these data indicate that increased miR-182 expression may promote cell proliferation, suppress the apoptotic pathway and ultimately confer aggressive traits on CRC cells.

**Electronic supplementary material:**

The online version of this article (10.1186/s12885-019-5982-9) contains supplementary material, which is available to authorized users.

## Background

MicroRNAs (miRNAs) regulate fundamental cellular processes, such as proliferation, differentiation, migration, angiogenesis and apoptosis, by repressing translation or inducing cleavage of their targets. MiRNAs are also involved in cancer development and progression, where they act as oncogenes or tumor suppressors [1]. A large variety of miRNAs have been shown to be involved, either as single elements or in combination [2], in the regulation of multiple tumorigenic processes and neoplastic phenotypes. In colorectal cancer (CRC), specific miRNA expression patterns were associated with tumor stage and other clinical parameters [3]. For instance, increased miR-21 expression in tumor tissue has been linked to decreased disease-free survival [4], and high miR-21 levels in plasma may be considered as a potential biomarker for the diagnosis of CRC [5]. Furthermore, up-regulation of miR-185, miR-221, miR-182, miR-17-3p, miR-34a, miR-106a, and down-regulation of miR-133b, miR-150, miR-378 (and combinations thereof), have been associated with cancer progression, recurrence and poor survival [6–12]. Moreover, miR-10b, miR-885-5p, miR-210, and miR-155 may provide predictive biomarkers of metastasis and recurrence [13, 14]. Differential response to chemotherapy has also been linked to miR-21, miR-320a, miR-150 and miR-129 expression levels [15–18].

In reference to CRC development, we identified miR-182-5p (miR-182) as one of the most up-regulated miRNAs in primary tumors compared to normal colon mucosa, thus suggesting its potential impact on target genes de-regulated in CRC [19]. A significant miR-182 increase is observed in the early phases of tumor development and is maintained in the metastatic process [20, 21]. Plasma miR-182 concentrations were higher in CRC patients at stage IV than in controls, and significantly decreased 1 month after radical hepatic metastasectomy, indicating that evaluation of circulating miR-182 may integrate the array of non-invasive blood-based monitoring and screening biomarkers [20].

miR-182 has been described as an oncogenic miRNA implicated in the development of various malignant histotypes by several studies (reviewed in [22]). In CRC, available evidence collectively indicates that miR-182 is one of the major players involved in the acquisition of malignant properties and it is associated with pro-proliferative signaling pathways and tumor invasion [23–25]. Nevertheless, the mechanisms underlying the ability of miR-182 to promote the tumorigenic process are not yet clarified. To fill this gap, we investigated the impact of miR-182 silencing in two human CRC cell lines endowed with different tumorigenic potential. Analysis of transcriptomic and in vitro readouts of miR-182 silencing indicated that this miRNA counteracts apoptosis and affects cell proliferation. In addition, the in vivo results showed that miR-182 sustains tumor growth by altering tumor cell cycle dynamics and morphology.

## Methods

### Cell lines and patients

HT-29, Caco2 and LoVo cells were obtained from the American Type Culture Collection (ATCC HTB-38, ATCC HTB-37, ATCC CCL-229). The CG-705, MICOL-S and MICOL-14^h-tert^ cell lines have been previously described [26] and were kindly provided by Dr. P. Dalerba (Columbia University, NY). Briefly, the CG-705 cell line was derived from a primary tumor of the right colon; MICOL-S cell line was derived from the hepatic metastasis of a primary right colon cancer; the MICOL-14^h-tert^ cell line was derived from a lymph-node metastasis of a patient with rectal cancer. MICOL-S and MICOL-14^h-tert^ cell lines have similar in vitro morphology and express the same differentiation markers, but they were derived from individuals with different primary cancer locations, as reported in Table 1 of the above quoted paper [26]. Both cell lines were unstable in vitro (i.e. they undergo growth arrest after a few in vitro passages) and were immortalized by h-TERT cDNA gene transfer. The MICOL-14^h-tert^ cell line behaves as non-tumorigenic in immunodeficient mice [27]. However, we demonstrated that the subcutaneous (s.c.) injection of MICOL-14^h-tert^ cell line into non-obese diabetic severe combined immunodeficient (NOD/SCID) mice in combination with angiogenic factors translated into the acquisition of an in vivo tumorigenic phenotype [27, 28]. This property was consistently maintained thereafter, and in vivo tumorigenesis experiments confirmed that MICOL-14^h-tert^ cells behaved as dormant, whereas NOD/SCID mice injected with the tumorigenic variant MICOL-14^tum^ developed aggressive tumors within 6 weeks (not shown). Authentication of specific genetic fingerprint by short tandem repeat (STR) DNA profile analysis showed that the two cell lines presented exactly the same *loci* number profile, and confirmed their genetic identity (data not shown); moreover, these cell lines were tested and scored negative for mycoplasma contamination when experiments were performed. All cell lines were grown in RPMI-1640 medium (Invitrogen, Milan, Italy) supplemented with 10% fetal bovine serum (FBS; Gibco, Invitrogen), L-glutamine, Pen/Strep and HEPES, and used within 6 months of thawing and resuscitation. The cells were harvested with trypsin-EDTA in their exponentially growing phase, and maintained in a humidified incubator at 37 °C with 5% CO_2_ in air. For this study, 5 patients with sporadic stage IV CRC were also selected [19], and their tumor tissue and normal mucosa samples were analyzed by qRT-PCR. The Ethics Committee of the University Hospital of Padova approved the study, and all patients provided written informed consent.

### RNA extraction, reverse transcription and quantitative RT-PCR analysis

RNA was extracted from cells 24, 48 and 72 h after their transfection using Trizol reagent (Thermo Fisher Scientific, MA), according to manufacturer’s instructions. RNA concentration and purity were measured with Nanodrop (Bio-Tek Instruments, Winooski, VT) and Agilent (Agilent Technologies, Santa Clara, CA). Reverse transcription and qRT-PCR experiments were conducted as previously described [19] using Taqman Gene Expression Assay (Applied Biosystem by Thermo Fisher Scientific). Expression data were normalized using as a reference RNU44 for miRNAs, and HPRT1 for transcripts.

### miRNA silencing by transient in vitro transfection

Cells were seeded in 6- or 24-well plates in complete RPMI medium for 24 h. The medium was then replaced with Opti-MEM® I Reduced Serum Medium (Thermo Fisher Scientific) and specific hsa-miR-182 mirVana™ miRNA inhibitor (Ambion by Thermo Fisher Scientific) was added to a total of 150 pmol/well; to allow cell transfection, Lipofectamine RNAiMAX transfection reagent (Invitrogen) was mixed with the miRNA inhibitor, according to protocol instructions. The mixture was incubated in the dark for 5 min at room temperature and then added to each well. In parallel, an equal number of cells were treated with an anti-miR-NC (mirVana™ miRNA inhibitor Negative Control #1; Ambion), as a control for data normalization of anti-mir-182-independent transfection effects. Cells plated in the medium used for the transfection, but without treatment, provided an additional control.

Moreover, to monitor inhibitor uptake efficiency by flow cytometry analysis, the same number of cells were transfected with a carboxyfluorescein-labeled RNA oligonucleotide (FAM™-labeled Anti-miR™ Negative Control; Ambion). After overnight incubation, the Opti-MEM medium supplemented with miRNA inhibitor or control was replaced with complete RPMI, and miRNA silencing was evaluated by qRT-PCR at different time points. At each time point, cells were also harvested to perform the experiments for miRNA function investigation. In all silencing experiments, transfection efficiency consistently exceeded 80%, and miRNA expression levels were decreased > 70% in transfected cells compared to controls.

### Apoptosis and cell cycle assay

To detect cell death, the Annexin-V-FLUOS staining kit (Roche, Mannheim, Germany) was used according to manufacturer’s instructions. For cell cycle analysis, cells were fixed with cold ethanol, stained with anti-human Ki67 (BD Biosciences, Franklin Lakes, NJ, USA) and then incubated for 1 h in a DAPI/RNAse solution. Cytofluorimetric analysis was performed on a FACS Calibur flow cytometer (Becton-Dickinson Immunocytometry Systems, NJ; excitation/emission wavelengths of 488/525 and 488/675 nm for Annexin-V and PI, respectively).

### Western blot analysis

Cell lysates were obtained in RIPA buffer containing protease inhibitor, and protein contents were quantified using Quantum Micro Protein Assay Kit (Euroclone, Milan, Italy). Experiments were conducted as previously described [29] using the following primary antibodies: rabbit anti-Cleaved Caspase-3 (1:1000; Cell Signaling Technology, MA), rabbit anti-PARP (1:1000; Cell Signaling Technology) and mouse anti-β-actin (1:1000; Santa Cruz Biotechnologies, CA). The following secondary antibodies were used: goat anti-rabbit (1∶5000; Bioss Antibodies, MA) or goat anti-mouse (1∶5000; Calbiochem MerckMillipore, Darmstadt, Germany) conjugated to horseradish peroxidase and visualized using Supersignal West Pico Chemiluminescent Substrate Kit (Thermo Fisher Scientific) with the Chemidoc XRS System and Quantity One 4.6.9 software (both from Bio-Rad, Hercules, CA). Densitometric analysis was performed with the ImageJ software (NIH).

### In vivo tumorigenesis assay

Non obese diabetic/severe combined immune deficiency (NOD/SCID) mice were bred in our SPF animal facility. All procedures involving animals and their care conformed to institutional guidelines that comply with national and international laws and policies (EEC Council Directive 86/609, OJ L 358, 12 December 1987). Before in vivo transfer, the tumorigenic MICOL-14^tum^ cells were treated with miR-182 inhibitor or anti-miR-NC as a control. For tumor establishment, 7 to 9-week-old mice were s.c. injected into both dorsolateral flanks with exponentially growing untreated or miR-182 silenced MICOL-14^tum^ cells (0.5 × 10^6^ cells in a 100 μl volume containing Matrigel). After 1 week, mirVana™ miR-182 inhibitor in vivo ready (Life Technologies by Thermo Fisher Scientific) or negative control were combined with Invivofectamine 2.0 Reagent (Life Technologies) and used for intratumoral injection to maintain in vivo miRNA silencing. The resulting tumor masses were inspected and measured as previously described [28]. In all experiments, the mice survived until the experimental endpoint, when they were sacrificed by cervical dislocation. Tumors were harvested by dissection, and either snap-frozen or fixed in formalin and embedded in paraffin for further analysis. Isofluran anaesthetic was used prior to injecting mice with tumor cells and before sacrifice.

### CRC grading and mitotic index evaluation

The tumor sections were evaluated by Hematoxylin and Eosin (H&E) staining for CRC grading and mitotic index evaluation. The 2010 WHO scoring for CRC Grading, based upon the percentage of gland formation (> 75%; 35–75% and < 35%, respectively), is as follows: G1 well differentiated cancer, G2 moderately differentiated cancer and G3 poorly differentiated cancer, and is. Main growth patterns were from less to more aggressive: glandular, trabecular and solid. The mitotic index, mirroring the ratio between the number of cells in a population undergoing and not undergoing mitosis, was calculated by counting the number of mitosis in 10 fields at 40X magnification.

### Gene expression analysis

Expression data were generated using the Affymetrix GeneChip PrimeView Human Gene Expression Array (Affymetrix by Thermo Fisher Scientific) using total RNA isolated from MICOL-14^h-tert^ and MICOL-14^tum^ cells transfected with either anti-miR-182 or anti-miR-NC. Raw data quality control was performed using the R package ‘affyQCreport’ [30]. Expression matrix reconstruction was obtained by ‘affy’ package [31] using RMA for data summarization and normalization. Transcript-level annotation of probesets, based on Ensembl (release 88), was obtained with R package ‘primeviewcdf’. Differential expression tests were conducted using Limma package [32], setting significance threshold to 0.05 for *p*-value, adjusted using FDR method for multiple testing correction.

Pathway enrichment analysis of differentially expressed genes was conducted using DAVID (Database for Annotation, Visualization and Integration Discovery, release 6.8) [33]. Significant GO terms, PIR keywords, and KEGG and Reactome pathways were selected considering adjusted *p*-values (Benjamini-Hochberg) at most 0.05. Experimentally validated and predicted miR-182 target transcripts were downloaded from MirTarBase (release 6.0) [34] and from TargetScanHuman (release 7.1) [35], respectively.

### Statistical analysis

Results were expressed as mean values ± SD. Two-tail Student’s t-test was performed on parametric groups. Values were considered significant at *p ≤ 0.05 and **p ≤ 0.01. All analyses were performed with SigmaPlot (Systat Software Inc. San Jose, CA).

## Results

### miR-182 is up-regulated in CRC cell lines and can be efficiently silenced in tumorigenic and non-tumorigenic cell lines

**Fig. 1:**
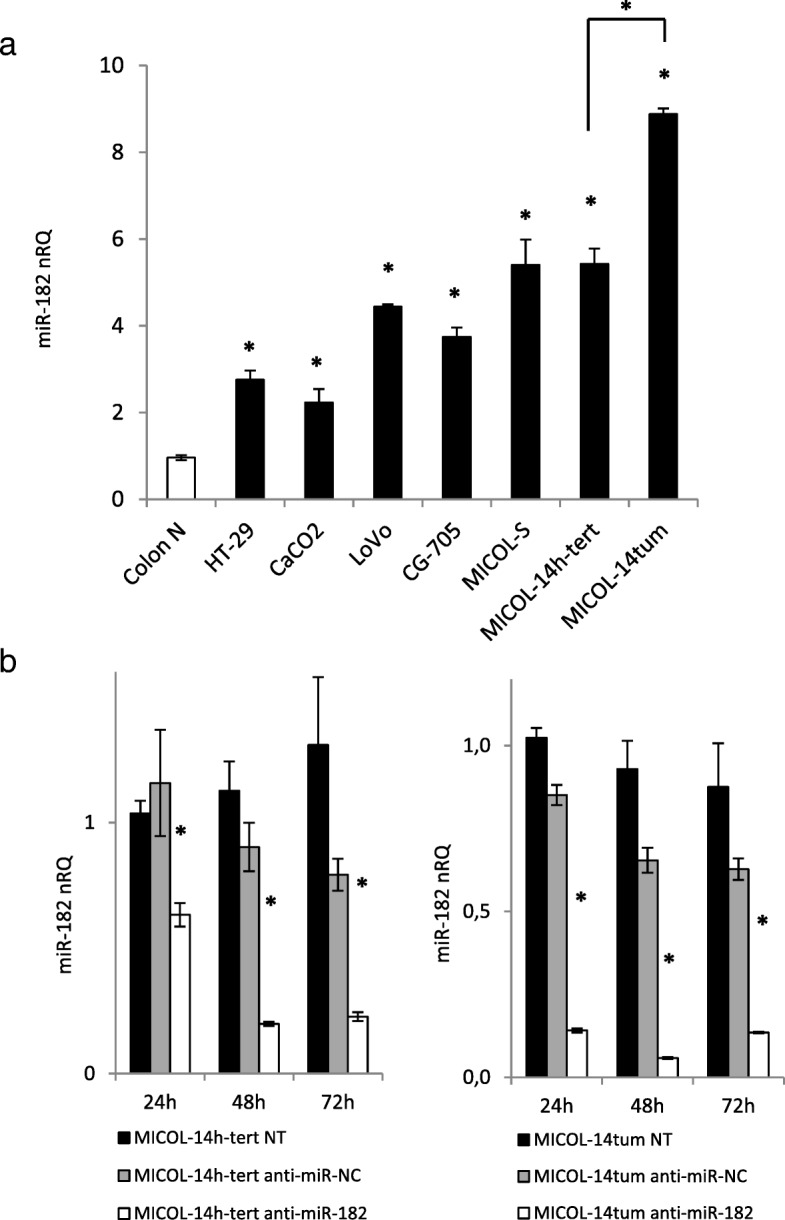
Expression of miR-182 in healthy colon mucosa and a panel of CRC cell lines**. a** The CRC cell lines were investigated by qRT-PCR for miR-182 expression levels compared to a pool of normal colon mucosa samples. All cell lines showed high levels of miR-182, and in particular in the tumorigenic variant MICOL-14^tum^ compared to MICOL14^h-tert^. Colon N, pool of normal colon mucosa. nRQ, normalized Relative Quantity. Mean values ± SD of 3 consecutive experiments are shown. *p < 0.01. **b** mir-182 inhibition in MICOL-14^h-tert^ and MICOL-14^tum^ cells. The evaluation of miR-182 expression levels was performed by qRT-PCR at 24, 48, and 72 h after the treatment. Data analysis was performed by ΔΔCt method, and the control groups (NT and anti-miR-NC treated cells) were used as a sample reference at each time point. Data were expressed as mean value ± SD of 3 independent experiments. nRQ, normalized Relative Quantity. *p < 0.01

miR-182 expression levels were evaluated by qRT-PCR in normal colon mucosa samples as a reference, and in a panel of seven CRC cell lines. Significant miR-182 up-regulation was observed in all the analyzed cancer cell lines (Fig. 1A), strengthening the evidence that increased miR-182 expression is a shared feature of CRC [19]. Highest miR-182 expression levels were measured in MICOL-14^tum^ cells followed by parental MICOL-14^h-tert^ cells. Based on these results, we focused subsequent experiments of miR-182 silencing in MICOL-14^tum^ and MICOL-14^h-tert^ cells, as a model of two cell lines which share the STR DNA profile but differ in key phenotypic properties such as the ability to generate tumors in immunodeficient recipients.

Treatment with anti-miR-182 effectively inhibited miR-182 expression in both cell lines. In particular, 24 h after treatment, the miR-182 expression resulted significantly repressed by a factor of 0.55 (*p* = 0.0034) and 0.17 (*p* = 0.0008) in MICOL-14^h-tert^ and MICOL-14^tum^, respectively. Silencing was maintained at all the time points considered and lasted for over 72 h in both cell lines (Fig. 1b).

### miR-182 silencing strongly increases apoptosis and affects cell cycle

**Fig. 2:**
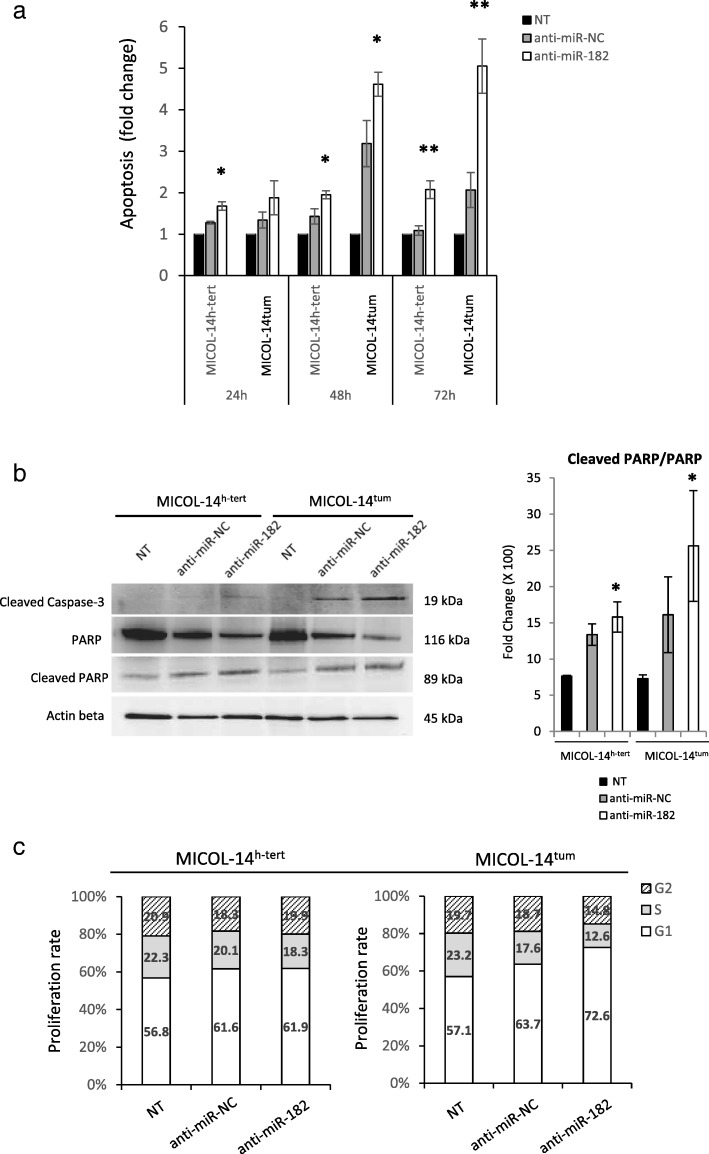
Effect of anti-miR-182 treatment on apoptosis and cell cycle progression of MICOL-14^h-tert^ and MICOL-14^tum^ cell lines. **a** miR-182 silencing was associated with increased sensitivity of cells to apoptosis in both MICOL-14^h-tert^ and MICOL-14^tum^ cell lines, as determined by Annexin V/PI staining at different time points following treatment. The results of three independent experiments in triplicate were expressed as mean fold change ± SD over the baseline apoptosis. **b** Western blot analysis (left panel) of cleaved Caspase-3 and PARP in MICOL-14^h-tert^ and MICOL-14^tum^ cell lines non-transfected (NT) and transfected with anti-miR-182 or control vector (miR-NC). The right panel shows the densitometric analysis of the ratio between cleaved and total PARP. β-actin was used as a loading control. The WB image is representative of three independent experiments; mean values ± SD of 3 consecutive experiments are shown in the right panel. **c** The cell cycle analysis was performed in MICOL-14^h-tert^ and MICOL-14^tum^ cell lines 48 h after treatment using Ki67 and DAPI staining. The control populations (NT and anti-miR-NC cells) were used as references at each time point. *p < 0.05

We next wondered whether miR-182 silencing could affect some key properties of MICOL-14^h-tert^ and MICOL-14^tum^ cells lines, such as apoptosis and cell cycle dynamics. Judging from annexin/PI staining, miR-182 inhibition was associated with a significant increase in apoptosis in both cell lines, compared to untreated cells (NT) and control anti-miR-NC treated cells (Fig. 2a). At 24 h post-treatment, the increase in apoptosis was comparable in MICOL-14^h-tert^ and MICOL-14^tum^ cells, whereas at later time points (48 and 72 h), apoptosis levels were significantly increased in the tumorigenic cell line compared to the dormant counterpart.

Western blot analysis of cleaved PARP and Caspase-3 proteins, performed 48 h post-treatment, confirmed the above results. Indeed, as shown in Fig. 2b, a decrease in total PARP and an eventual increase in cleaved PARP was observed in both MICOL-14^h-tert^ and MICOL-14^tum^ cells, compared to the cells treated with control anti-miR-NC. However, the ratio between total and cleaved PARP was lower in MICOL-14^tum^ cells, indicating that the complex machinery regulating apoptotic phenomena was preferentially affected by miR-182 silencing in the tumorigenic cell line.

The involvement of miR-182 in cell cycle progression was supported by proliferation rate analysis. While MICOL-14^h-tert^ cells only disclosed minimal changes in cell cycle profile after anti-miR-182 treatment (Fig. 2c), a significant increase in the fraction of cells in G0/G1 phases was observed in MICOL-14^tum^ cells, associated with a corresponding decrease in the S and G2 phases (Fig. 2c). These data indicated that miR-182 inhibition in MICOL-14^tum^ cells may modulate cell proliferation rate and strongly induce apoptosis.

### miR-182 silencing significantly affects gene expression profile of MICOL-14^h-tert^ and MICOL-14^tum^ cells

To explore the complex biological processes involved in the above-described functional changes, transcript and gene expression profiling was performed on MICOL-14^h-tert^ and MICOL-14^tum^ 24 h after treatment with anti-miR-182 or anti-miR-NC. Four replicates for cell type and condition were tested. Expression profiles of 49,293 probesets, corresponding to 41,532 transcripts and to 19,942 individual genes, in the 16 samples considered, were acquired.

**Fig. 3:**
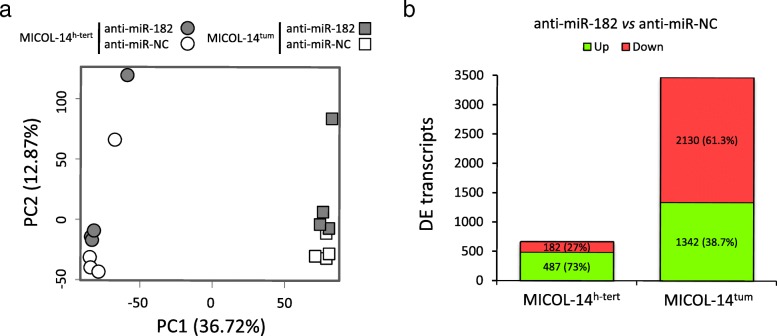
Gene expression profiles changes associated with miR-182 silencing in MICOL-14^h-tert^ and MICOL-14^tum^ cells**. A.** Principal Component Analysis (PCA) of samples according to transcript expression profiles measured by Primeview array analysis indicates differences among control samples of different cell lines and more importantly, for each cell line, a clear separation of anti-miR-182 treated and control samples pointed toward the readout of miR-182 silencing. **B.** Number of significantly up- or down-regulated transcripts differentially expressed is compared between anti-miR-182-treated and control samples in MICOL-14^h-tert^ and MICOL-14^tum^ cells

Unsupervised Principal Component Analysis (PCA) of transcript expression profiles showed that samples separated first for cell line, indicating that the two cell lines display highly different expression profiles, and then by treatment, underlying the effect of miR-182 inhibition on expression profiles of both lines (Fig. 3a). Accordingly, expression data informed on differential expression between the dormant and the tumorigenic cell lines and, more importantly, on expression changes determined by miR-182 silencing in each cell line.

Comparing anti-miR-182 vs anti-miR-NC, significant differential expression was detected in both cell lines (Fig. 3b), with a more marked impact of miR-182 silencing in MICOL-14^tum^ (3472 differentially expressed transcripts from 1382 genes, 40% up-regulated), than in MICOL-14^h-tert^ cells (669 transcripts from 243 genes, 73% up-regulated). Genes differentially expressed after miR-182 silencing are expected to include both direct miRNA targets, likely enriched with those up-regulated after miRNA silencing, and indirectly regulated genes due to miR-182 impact on transcriptional and post-transcriptional regulators in complex regulatory circuits.

According to our data, 759 genes had transcripts (1825 in total) significantly up-regulated after miR-182 inhibition in one or both cell lines. Notably, 15 of these genes (*ATF1, PNISR, ANKRD36, ARRDC3, NR3C1, ZFP36L1, RGS2, DDAH1, SESN2, FLOT1, FAM193A, BRWD1, RBM12, QSER1, TNRC6A*) were already validated as canonical miR-182 targets according to miRTarBase. Upregulated transcripts from additional 234 genes were TargetScan*-*predicted miR-182 targets (Additional file 1: Table S1). Of the 158 genes with transcripts differentially expressed after miR-182 inhibition in both cell lines, a vast majority (153) showed expression changes in the same direction in the two cell lines, prevalently (103) up-regulation.

Functional Gene Ontology (GO) terms and significantly enriched pathways were detected considering genes differentially expressed after miR-182 inhibition in each cell line Additional files 2 and 3: Tables S2-S3) and in both cell lines (Table 1). According to in vitro data on the impact of miR-182 silencing on the apoptotic process, “positive regulation of apoptotic process” was the most enriched biological process among genes differentially expressed in both cell lines after miR-182 inhibition. Moreover, an enrichment of p53 signaling and FoxO signaling pathways, both multifunctional processes in the cross-talk with apoptosis regulation through common genes and proteins [36], was also observed.

**Table 1:** Gene Ontology (GO) functional terms and KEGG pathways significantly enriched considering 158 genes differentially expressed after miR-182 inhibition in both cell lines. BP, Biological Process; CC, Cellular Component; MF, Molecular Function

Functional category	Term/Pathway	Gene symbol	Genes	Fold Enrich.	Adj. *p*-value
GO BP	Regulation of transcription, DNA-templated	ITGB3BP, EID3, SRSF10, EID2B, PPHLN1, ZNF557, SPTY2D1, NR3C1, ZNF638, ZNF655, ZNF165, ZFP36L1, SRRT, SFSWAP, ZNF181, ZNF226, HIF1A, PNRC2, THAP1, TCF3, NFIA, ZNF267, ZNF101	23	2.52	0.027
	Positive regulation of apoptotic process	ITGB3BP, HIF1A, SQSTM1, TRIO, GADD45B, VAV2, GADD45A, LATS1, BCL2L11, IP6K2, PHLDA1	11	5.21	0.0333
GO CC	Nucleus	ITGB3BP, TUBB2A, EID2B, CLK1, HIST2H4A, TCEAL1, CAMKK2, NFATC2IP, FUBP1, SFSWAP, CCNE1, ZNF181, BLZF1, CLK4, ANKRD11, NSMCE2, AKIRIN1, IP6K2, ZNF101, TIGD1, RELB, CCNL1, NABP1, HIF1A, MSANTD4, CUX1, GADD45B, GADD45A, SRSF10, SLF2, ZNF557, NR3C1, ZNF655, PXK, SESN2, TSPYL4, ZFP36L1, SFR1, VRK2, ZNF226, HIST1H4E, THAP1, TCF3, ZNF267, FKTN, TKT, ZNF165, RERG, CDKN1A, ATF3, ZBED4, PNRC2, RNPC3, PDCD6, PPP2R3C, NFIA	56	1.53	0.0202
	Nucleoplasm	ITGB3BP, EID3, SRSF10, NR3C1, ZNF638, HIST2H4A, TCEAL1, FUBP1, CCNE1, SRRT, BLZF1, SQSTM1, ANKRD11, HIST1H4E, NSMCE2, AKIRIN1, TCF3, AKT3, IP6K2, NQO2, PPP4R3B, PPHLN1, RELB, TKT, TRNT1, NABP1, CDKN1A, ATF3, HIF1A, SMARCC1, MAPK9, RNPC3, SCAF8, CUX1, GADD45A, NFIA	36	1.94	0.0127
GO MF	Protein binding	ITGB3BP, TUBB2A, CLK1, HIST2H4A, LATS1, RSRC2, FUBP1, SFSWAP, CCNE1, BLZF1, CLK4, ARL14, RABGEF1, NSMCE2, AKIRIN1, AKT3, ZNF101, NQO2, IP6K2, RAP2A, TTC32, RELB, CCNL1, RBKS, CCT6A, C8ORF44-SGK3, MRM1, BCL2L11, NABP1, HIF1A, NUCB2, USO1, MAPK9, G0S2, MAPRE2, GADD45B, SCAF8, GADD45A, EID3, SRSF10, SLC38A9, SNX5, CALD1, SLF2, RPS15A, FAM122A, FKBP1A, NR3C1, C6ORF226, ZNF655, TSPYL4, PPCDC, SESN2, ZFC3H1, ZFP36L1, SRRT, SFR1, VRK2, C1ORF50, KLC1, SQSTM1, HIST1H4E, LETMD1, THAP1, TCF3, INPP5A, PHLDA1, CCNB1IP1, RBM12B, PPHLN1, ASXL1, TRIO, TKT, RCAN3, VAV2, SGTB, ATG3, RPL28, ZNF165, PPIF, CDKN1A, C1ORF116, ATF3, SMARCC1, PNRC2, ZBED4, RIT1, AGR2, PDCD6, ALG13, PPP2R3C	91	1.46	1.05E-05
KEGG	FoxO signaling pathway	CDKN1A, MAPK9, GADD45B, C8ORF44-SGK3, GADD45A, AKT3, BCL2L11	7	8.31	0.0185
	p53 signaling pathway	CCNE1, CDKN1A, GADD45B, SESN2, GADD45A	5	11.86	0.0434

**Fig. 4:**
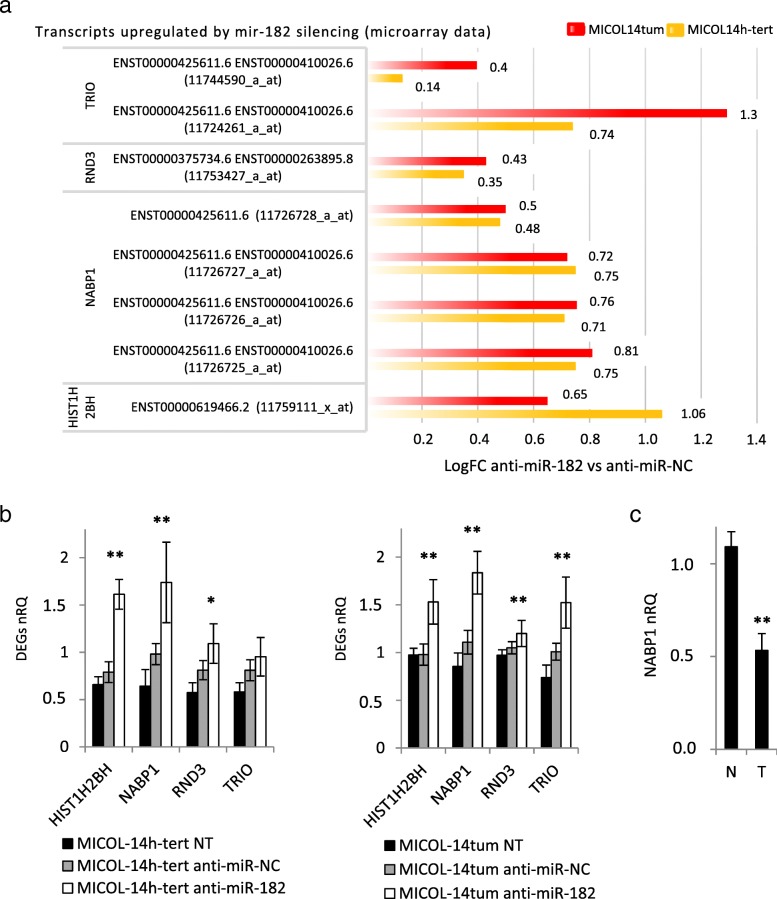
Description and qRT-PCR evaluation of predicted transcript targets after miR-182 silencing**. a** Microarray analysis in MICOL-14 h-tert and MICOL-14tum cell lines showed upregulation of miR-182 target gene transcripts after miR-182 inhibition (positive logFC comparing anti-miR-182 vs anti-miR-NC). **b** qRT-PCR evaluation of the transcript expression levels of selected genes in MICOL-14^h-tert^ and MICOL-14^tum^ cell lines. Data analysis was performed by ΔΔCt method, and the control groups (NT and anti-miR-NC treated cells) were used as sample references in cell lines. Data were expressed as mean values ± SD of three independent experiments. nRQ, normalized Relative Quantity. *p < 0.05 **p < 0.01. **c** NABP1 levels were compared in a pool of primary CRC samples (T), in which increased miR-182 levels were known, and matched normal colon mucosa (N)

The significant upregulation after miR-182 silencing of miR-182 predicted target transcripts of *HIST1H2BH*, *NABP1*, *RND3*, and *TRIO* genes (all encoding proteins with potential role in DNA-damage response and invasion) was confirmed by transcript-specific qRT-PCR assay (Fig. 4a-b, and Additional file 4: Table S4). In particular, the *NABP1* gene, which is involved in the GO “DNA repair” pathway taking part in the apoptotic process, was significantly enriched in the anti-miR-182-treated tumorigenic cell line. Interestingly, a significant NABP1 expression decrease was observed in a pool of primary CRC samples, in which increased miR-182 levels were previously assessed [21], compared to matched normal colon mucosa (Fig. 4c).

### miR-182 inhibition in MICOL-14^tum^ xenografts impairs in vivo tumor growth and is associated with morphological and histological changes

**Fig. 5:**
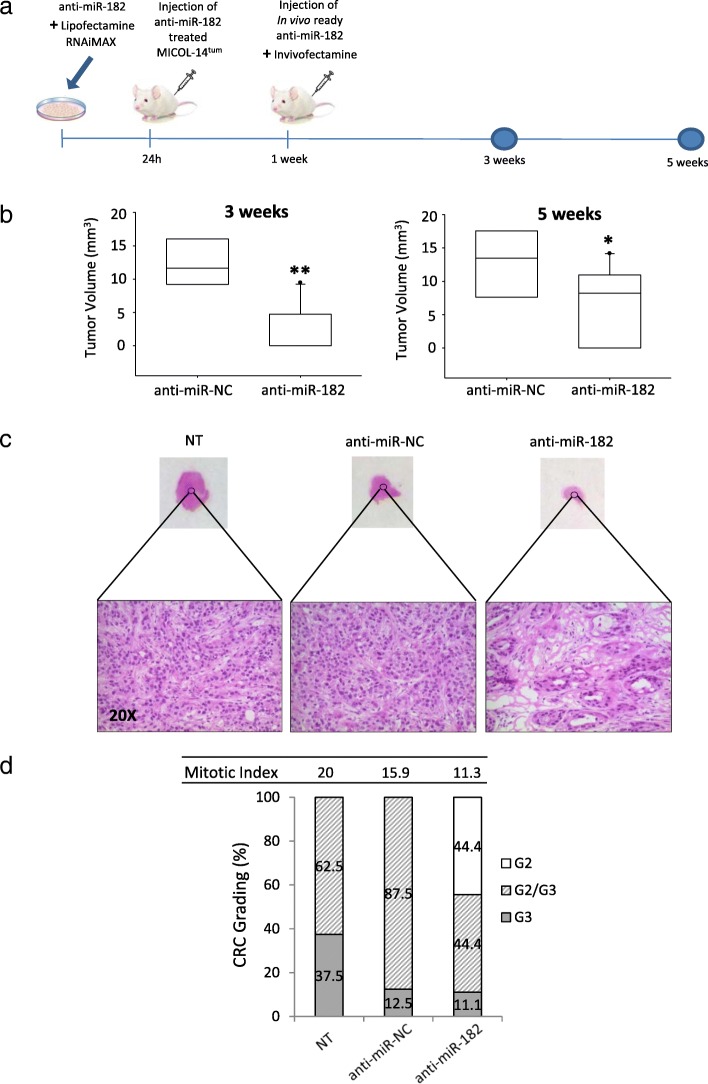
Effect of miR-182 silencing on tumor outgrowth and histological features of MICOL-14^tum^ xenografts. **a** Experimental layout for the study of the effects of miR-182 silencing on the ability of MICOL-14^tum^ cells to generate tumors upon injection into immunodeficient hosts. MICOL-14^tum^ cells were treated with anti-miR-182 or anti-miR-NC, and after 24 h they were s.c. injected into NOD/SCID mice. A week later, an intratumoral injection of in vivo ready anti-miR-182 and Invivofectamine was performed to sustain miR-182 knockdown. **b** Tumor outgrowth was measured 3 and 5 weeks after inoculation of MICOL-14^tum^. The control group (anti-miR-NC treated cells) was used as a reference at each time point. Center lines of box plots show the medians; box limits indicate the 25th and 75th percentiles, as determined by R software. *p < 0.05, **p < 0.01. **c** Reduction of tumor growth and changes of the morphological features of miR-182-silenced MICOL-14^tum^ xenografts. H&E staining of tumor sections is shown at the bottom. Magnification 20X. The control groups (NT and anti-miR-NC treated cells) were used as a reference. **d** Mitotic index and grading in tumor masses obtained from anti-miR-182-treated MICOL-14^tum^. Control cells (NT and anti-miR-NC) mostly grew as G2/G3 or G3 adenocarcinomas, whereas anti-miR-182 masses mainly showed a moderately differentiated adenocarcinoma profile (G2 and G2/G3)

In vitro analyses and gene expression profiles strongly supported a role of miR-182 in the MICOL-14^tum^ cells tumorigenic phenotype. Thus, we investigated whether miR-182 silencing could also affect the in vivo tumor growth of MICOL-14^tum^ cells in a xenogeneic model of tumorigenesis. To this end, MICOL-14^tum^ cells were treated with ant-miR-182 or the appropriate control, and injected s.c into NOD/SCID mice. Although the in vitro silencing effect of anti-miR-182 was still present in MICOL-14^tum^ cells several days after treatment (see Fig. 1b, and data not shown), 1 week after cell transfer an intra-tumor injection of anti-miR-182 was performed to buttress in vivo miR-182 silencing (Fig. 5a). The mice inoculated with control MICOL-14^tum^ cells developed significantly larger tumors, compared to mice injected with anti-miR-182-treated cells (Fig. 5b). Interestingly, miR-182 inhibition was associated with a significant reduction in tumor size 3 weeks after injection (*p* = 1.56 × 10^− 5^), and 5 weeks later the volume of tumor masses was still significantly different (Fig. 5b; *p* = 0.037).

Notably, miR-182 inhibition was associated with evident histological and morphological changes in the tumor tissue harvested from immunodeficient recipients (Fig. 5c). In fact, the tumor masses generated by MICOL-14^tum^ control cells consistently showed moderately to poorly differentiated adenocarcinoma with bulky appearance, trabecular-solid pattern, minimal fibrosis and pushing borders. In contrast, the tumor masses developed after inoculation of anti-miR-182-treated MICOL-14^tum^ cells showed mainly moderately differentiated adenocarcinoma with mild fibrosis within (Fig. 5c). Moreover, the average mitotic index of tumor masses was significantly higher in control mice than in animals injected with anti-miR-182-treated cells (Fig. 5d), indicating that miR-182 inhibition also impairs cell proliferation in vivo.

## Discussion

miR-182 deregulation has been reported in several human cancer types, including CRC. We previously observed that miR-182 overexpression is already present in the transition from normal colonic mucosa to tubular adenoma and is stably maintained in primary CRC tumor and liver metastases. This seems to indicate that the miR-182 upregulation occurs in early premalignant development and is associated with the maintenance of the malignant phenotype [19]. Furthermore, we also demonstrated that high expression levels of miR-182 do not characterize mucosa samples from patients with inflammatory bowel disease, thus suggesting that its deregulation is not a mere consequence of the chronic inflammatory process [21]. Interestingly, in a large functional miRNA screening, Cekaite et al. found that *miR-182* gene, a component of miRNA cluster miR-183-96-182 located in 7q32 genomic region, is amplified in 26% of primary CRC and 30% of liver metastases [25]. In the same large-scale analysis, a link between reduced apoptosis and deregulation of a combined set of miRNAs, namely miR-9, − 31, and − 182, was also reported in two independent CRC cell lines, suggesting that miR-182 is involved in CRC development and progression by promoting cell survival. Thus, the impact of miR-182 on apoptosis, proliferation and invasion as well as on chemo-resistance has recently been addressed in search for a link between its high expression and the acquisition of functional properties favorable to tumor development [37–39].

In the present study, the impact of miR-182 silencing on the biological properties of MICOL-14^h-tert^ and MICOL-14^tum^ cell lines was investigated in vitro and in vivo, demonstrating that miR down-regulation strongly increases apoptosis and affects cell cycle dynamics in both cell lines, with a more pronounced and long-lasting effect in the tumorigenic cell line compared to the dormant counterpart. Evidence that anti-miR-182 treatment impairs the tumorigenic potential of the MICOL-14^tum^ cell line after the xenogenic transplant in immunodeficient mice was also provided. However, miR-182 silencing was associated with a delay in the generation of tumors by the MICOL-14^tum^ cell line and did not abrogate its tumorigenic potential. Reactivation of miR-182 a few weeks after silencing in some transduced cells, and their eventual outgrowth, or the presence within the transferred population of a few cells with ineffective silencing could explain this finding.

miRNAs are highly pleiotropic and a single miRNA can influence many genes. Thereby deregulation of a single miRNA can deeply affect cellular phenotypes. Indeed, tumor masses generated by miR-182 silenced MICOL-14^tum^ cells showed histological features compatible with less aggressive carcinomas, compared to untreated tumors. This could suggest that miR-182 may play a role in apoptosis as well as in other processes, including cell survival and differentiation. On the other hand, gene expression profiling showed that miR-182 silencing affects the expression of a large number of genes in both MICOL-14^h-tert^ and MICOL-14^tum^ cells, with a stronger impact in the tumorigenic cell line. The two cell lines were endowed with different gene expression profiles and in response to anti-miR-182 treatment, behaved differently. Nevertheless, 158 genes were differentially expressed in both cell lines and pointed to three significantly enriched pathways correlated with cellular survival: “positive regulation of apoptotic process”, “p53 signaling” and “FoxO signaling”. These pathways shared two interesting components of the *Gadd* gene family, *Gadd 45A/B.* Gadd protein expression can be induced, in a p53-dependent or –independent way, by DNA damage and other stress signals associated with growth arrest and apoptosis [40]. These proteins have been implicated in a variety of responses to cell injury, including the control of cell cycle checkpoints, apoptosis, and DNA repair. We confirmed by qRT-PCR assay the significant upregulation after miR-182 silencing of two genes, *HIST1H2BH and NABP1. HIST1H2BH* is a member of a large histone gene family, histones H2A, H2B, H3 and H4. Two heterodimers of H2A/H2B and one H3/H4 tetramer, associated with DNA, form the compact structure of chromatin in nucleosome. Interestingly, H2A/H2B plays an important role in processes that allow for transcription, DNA replication and DNA repair [41]. *NABP1,* also known as *SSBP2*, encodes a component of the single-strand DNA binding complex, whose role in the maintenance of genomic stability has only recently emerged [42]. *NABP1* influences diverse endpoints in the cellular DNA damage response, including cell cycle checkpoint activation. We demonstrated in a pool of primary CRC samples the significant decrease of NABP1 mRNA levels in tumor tissue compared to normal mucosa, strengthening observations on gene expression. Our findings are in line with data by Krishnan et al. in breast cancer [37], and specifically support the idea that, in CRC as well, miR-182-mediated deregulation of the DNA damage response pathway could translate into impaired DNA repair with downstream effects on genetic stability and cellular transformation.

## Conclusions

Altogether, our data highlight the relevance of miR-182 dysregulation in CRC tumorigenesis and provide evidence that this miRNA controls apoptosis and proliferation, clearly pointing to specific components of apoptosis and DNA repair processes highly represented in the network of miR-182 validated or predicted target genes.

## Additional files

Additional file 1: **Table S1.** Predicted and validated miR-182 targets upregulated after miR-182 silencing in one or both cell lines. Only transcripts with average expression at least 3, significantly up-regulated with a log FC > 0.3 are reported (val, validated target according to MiRTarBase; pre, TargetScan predicted target). (DOCX 148 kb)

Additional file 2: **Table S2.** Gene Ontology (GO) functional terms, KEGG and Reactome pathways significantly enriched considering 242 genes differentially expressed after miR-182 inhibition in MICOL-14^h-tert^ cells. BP, Biological Process; CC, Cellular Component; MF, Molecular Function. (DOCX 30 kb)

Additional file 3: **Table S3.** Gene Ontology (GO) functional pathways significantly enriched considering 1382 genes differentially expressed after miR-182 inhibition in MICOL-14^tum^ cells. BP, Biological Process; CC, Cellular Component; MF, Molecular Function. (DOCX 25 kb)

Additional file 4: **Table S4.** MiR-182 predicted target transcripts for which differentially expression in MICOL-14^h-tert^ and/or MICOL-14^tum^ cells after treatment was confirmed by RT-PCR. The table showed the transcripts and the correspondinggenes, probesets and Taqman Assay ID used for experimental qRT-PCR validation. For each probeset and cell line, the expression variation observed according to Primeview Microarray data analysis is shown as LogFC of the anti-miR-182 vs anti-miR-NC comparison; values corresponding to a stastistically significant differential expression are in bold. (DOCX 19 kb)

## Data Availability

The datasets obtained and/or analyzed during the current study are available from the corresponding author upon reasonable request.

## Abbreviations

CRC: Colorectal cancer
miRNA: microRNA
NOD/SCID: Non obese diabetic/severe combined immune deficiency
s.c.: Subcutaneous
