# Supplementary material for: Silencing of miR-182 is associated with modulation of tumorigenesis through apoptosis induction in an experimental model of colorectal cancer
Source: BMC Cancer. 2019 Aug 20;19:821. doi: 10.1186/s12885-019-5982-9 (PMC6700772; doi:10.1186/s12885-019-5982-9)
Supplement: Supplementary file 1 — Table S1. Predicted and validated miR-182 targets upregulated after miR-182 silencing in one or both cell lines. Only transcripts with average expression at least 3, significantly up-regulated with a log FC > 0.3 are reported (val, validated target according to MiRTarBase; pre, TargetScan predicted target). (DOCX 148 kb) [file 12885_2019_5982_MOESM1_ESM.docx]

**Supplementary Table 1.** **Predicted and validated miR-182 targets upregulated after miR-182 silencing in one or both cell lines.** Only transcripts with average expression at least 3, significantly up-regulated with a log FC > 0.3 are reported (val, validated target according to MiRTarBase; pre, TargetScan predicted target).

| **Probeset** | **Ensembl Transcript** | **RefSeq Transcript** | **Gene Symbol** | **Gene Description** | **MICOL-14tum** | | **MICOL-14h-tert** | | **miR-182 target** |
| --- | --- | --- | --- | --- | --- | --- | --- | --- | --- |
|  |  |  |  |  | **LogFC anti-miR-182 vs anti-miR-NC** | **Average Expression** | **LogFC anti-miR-182 vs anti-miR-NC** | **Average Expression** |  |
| 11753238_x_at | ENST00000357042.4 | NM_001164315, NM_198555, XM_006712514, XM_006712516, XM_011511130, XM_011511131, XM_011511132, XM_011511133, XM_011511134, XM_011511135, XM_011511136, XM_011511137, XM_011511138, XM_011511139, XM_011511140, XM_011511141, XM_011511142, XM_011511143, XM_011511144, XM_011511145, XR_427086, XR_922917, XR_922918, XR_922919, XR_922920, XR_922921, XR_922922, XR_922923, XR_922924, XR_922925 | ANKRD36 | Ankyrin repeat domain 36 | 0.54 | 6.28 | 0.08 | 6.28 | val |
| 11762617_x_at | ENST00000357042.4 | NM_001164315, NM_198555, XM_006712514, XM_006712516, XM_011511130, XM_011511131, XM_011511132, XM_011511133, XM_011511134, XM_011511135, XM_011511136, XM_011511137, XM_011511138, XM_011511139, XM_011511140, XM_011511141, XM_011511142, XM_011511143, XM_011511144, XM_011511145, XR_427086, XR_922917, XR_922918, XR_922919, XR_922920, XR_922921, XR_922922, XR_922923, XR_922924, XR_922925 | ANKRD36 | Ankyrin repeat domain 36 | 0.49 | 6.08 | 0.09 | 6.08 | val |
| 11718723_at | ENST00000265138.3 | NM_020801, XR_948281 | ARRDC3 | Arrestin domain containing 3 | 0.41 | 7.71 | 0.24 | 7.71 | val |
| 11743163_at | ENST00000262053.3 | NM_005171, XM_011538386, XM_011538387, XM_011538388 | ATF1 | Activating transcription factor 1 | 0.25 | 8.07 | 0.31 | 8.07 | val |
| 11751694_a_at | ENST00000262053.3 | NM_005171, XM_011538386, XM_011538387, XM_011538388 | ATF1 | Activating transcription factor 1 | 0.41 | 8.52 | 0.38 | 8.52 | val |
| 11728438_a_at | ENST00000342449.3 | NM_001007246, NM_018963, NM_033656, XM_011529611, XM_011529612, XM_011529613 | BRWD1 | Bromodomain and WD repeat domain containing 1 | 0.49 | 6.08 | 0.32 | 6.08 | val |
| 11716917_a_at | ENST00000535924.2 | NM_001134445, NM_012137, XM_005270707, XM_005270709, XM_005270710, XM_006710544, XM_011541158 | DDAH1 | Dimethylarginine dimethylaminohydrolase 1 | 0.50 | 6.50 | 0.41 | 6.50 | val |
| 11748534_a_at | ENST00000535924.2 | NM_001134445, NM_012137, XM_005270707, XM_005270709, XM_005270710, XM_006710544, XM_011541158 | DDAH1 | Dimethylarginine dimethylaminohydrolase 1 | 0.54 | 6.24 | 0.41 | 6.24 | val |
| 11757504_a_at | ENST00000535924.2 | NM_001134445, NM_012137, XM_005270707, XM_005270709, XM_005270710, XM_006710544, XM_011541158 | DDAH1 | Dimethylarginine dimethylaminohydrolase 1 | 0.32 | 9.41 | 0.35 | 9.41 | val |
| 11746034_a_at | ENST00000505311.1 | NM_001256666, NM_001256667, NM_001256668, NM_003704, NR_046335, NR_046336, XM_006713930, XM_006713932, XM_011513590, XM_011513591, XM_011513592, XM_011513593 | FAM193A | Family with sequence similarity 193, member A | 0.43 | 5.92 | 0.09 | 5.92 | val |
| 11743047_x_at | ENST00000376389.3 | NM_005803, XM_005248780, XM_005248781, XM_005272759, XM_005272760, XM_005274909, XM_005274910, XM_005275335, XM_005275336, XM_005275502, XM_005275503, XM_006714947, XM_006725465, XM_006725672, XM_006725971, XM_006726072 | FLOT1 | Flotillin 1 | 0.33 | 9.80 | 0.22 | 9.80 | val |
| 11748081_a_at | ENST00000376389.3 | NM_005803, XM_005248780, XM_005248781, XM_005272759, XM_005272760, XM_005274909, XM_005274910, XM_005275335, XM_005275336, XM_005275502, XM_005275503, XM_006714947, XM_006725465, XM_006725672, XM_006725971, XM_006726072 | FLOT1 | Flotillin 1 | 0.40 | 8.06 | 0.24 | 8.06 | val |
| 11748082_x_at | ENST00000376389.3 | NM_005803, XM_005248780, XM_005248781, XM_005272759, XM_005272760, XM_005274909, XM_005274910, XM_005275335, XM_005275336, XM_005275502, XM_005275503, XM_006714947, XM_006725465, XM_006725672, XM_006725971, XM_006726072 | FLOT1 | Flotillin 1 | 0.35 | 8.83 | 0.23 | 8.83 | val |
| 11743738_a_at | ENST00000394464.2 | NM_000176, NM_001018074, NM_001018075, NM_001018076, NM_001018077, NM_001020825, NM_001024094, NM_001204258, NM_001204259, NM_001204260, NM_001204261, NM_001204262, NM_001204263, NM_001204264, NM_001204265, XM_005268419, XM_005268420, XM_005268422, XM_005268423, XM_011537636, XM_011537637 | NR3C1 | Nuclear receptor subfamily 3, group C, member 1 (glucocorticoid receptor) | 0.33 | 7.72 | 0.28 | 7.72 | val |
| 11743739_a_at | ENST00000394464.2 | NM_000176, NM_001018074, NM_001018075, NM_001018076, NM_001018077, NM_001020825, NM_001024094, NM_001204258, NM_001204259, NM_001204260, NM_001204261, NM_001204262, NM_001204263, NM_001204264, NM_001204265, XM_005268419, XM_005268420, XM_005268422, XM_005268423, XM_011537636, XM_011537637 | NR3C1 | Nuclear receptor subfamily 3, group C, member 1 (glucocorticoid receptor) | 0.21 | 7.42 | 0.37 | 7.42 | val |
| 11751289_a_at | ENST00000394464.2 | NM_000176, NM_001018074, NM_001018075, NM_001018076, NM_001018077, NM_001020825, NM_001024094, NM_001204258, NM_001204259, NM_001204260, NM_001204261, NM_001204262, NM_001204263, NM_001204264, NM_001204265, XM_005268419, XM_005268420, XM_005268422, XM_005268423, XM_011537636, XM_011537637 | NR3C1 | Nuclear receptor subfamily 3, group C, member 1 (glucocorticoid receptor) | 0.22 | 8.05 | 0.40 | 8.05 | val |
| 11744502_a_at | ENST00000369239.5 | NM_015491, NM_032870, XM_005266912, XM_005266913, XM_005266914, XM_005266915, XM_005266916, XM_005266917 | PNISR | PNN-interacting serine/arginine-rich protein | 0.40 | 8.24 | 0.14 | 8.24 | val |
| 11755634_x_at | ENST00000369239.5 | NM_015491, NM_032870, XM_005266912, XM_005266913, XM_005266914, XM_005266915, XM_005266916, XM_005266917 | PNISR | PNN-interacting serine/arginine-rich protein | 0.32 | 9.53 | 0.11 | 9.53 | val |
| 11720906_a_at | ENST00000399302.2 | NM_001076786, NM_024774, XM_006718323, XM_006718325 | QSER1 | Glutamine and serine rich 1 | 0.35 | 7.48 | -0.05 | 7.48 | val |
| 11719925_a_at | ENST00000374114.3 | NM_001198838, NM_001198840, NM_006047, NM_152838 | RBM12 | RNA binding motif protein 12 | 0.23 | 9.33 | 0.44 | 9.33 | val |
| 11715757_a_at | ENST00000235382.5 | NM_002923 | RGS2 | Regulator of G-protein signaling 2 | 0.40 | 7.10 | 0.16 | 7.10 | val |
| 11718324_s_at | ENST00000253063.3 | NM_031459, XR_946773 | SESN2 | Sestrin 2 | 0.62 | 8.06 | 1.12 | 8.06 | val |
| 11718325_at | ENST00000253063.3 | NM_031459, XR_946773 | SESN2 | Sestrin 2 | 0.64 | 8.79 | 1.12 | 8.79 | val |
| 11717817_a_at | ENST00000395799.3 | NM_014494, XM_005255254, XM_005255257, XM_006721039, XM_011545791, XM_011545792, XM_011545793, XM_011545794, XM_011545795, XM_011545796 | TNRC6A | Trinucleotide repeat containing 6A | 0.39 | 9.17 | 0.40 | 9.17 | val |
| 11755871_s_at | ENST00000395799.3 | NM_014494, XM_005255254, XM_005255257, XM_006721039, XM_011545791, XM_011545792, XM_011545793, XM_011545794, XM_011545795, XM_011545796 | TNRC6A | Trinucleotide repeat containing 6A | 0.41 | 8.37 | 0.40 | 8.37 | val |
| 11726889_at | ENST00000555997.1 | NM_001244698, NM_001244701, NM_004926 | ZFP36L1 | ZFP36 ring finger protein-like 1 | 1.02 | 6.54 | 1.56 | 6.54 | val |
| 11721112_a_at | ENST00000369238.6 | NM_016361, XM_011509601, XR_921820 | ACP6 | Acid phosphatase 6, lysophosphatidic | 0.35 | 8.29 | 0.29 | 8.29 | pre |
| 11745589_a_at | ENST00000295903.4 | NM_182920, XM_005265329, XR_245151 | ADAMTS9 | ADAM metallopeptidase with thrombospondin type 1 motif 9 | 0.35 | 5.25 | 0.10 | 5.25 | pre |
| 11731981_a_at | ENST00000414691.3 | NM_001143948, NM_032744, XM_005249454, XM_005249455, XM_011514956, XM_011514958, XM_011514959, XR_926322 | ADTRP | Androgen-dependent TFPI-regulating protein | 0.41 | 5.29 | 0.06 | 5.29 | pre |
| 11730312_a_at | ENST00000394457.3 | NM_152392, XM_006711943, XM_011532541, XM_011532542, XM_011532543, XM_011532544, XM_011532545, XM_011532546, XM_011532547, XR_939658, XR_939659, XR_939660 | AHSA2 | AHA1, activator of heat shock 90kda protein atpase homolog 2 (yeast) | 0.46 | 7.42 | 0.35 | 7.42 | pre |
| 11763810_a_at | ENST00000025301.2 | NM_016248, NM_144490, XM_005266247, XM_005266248, XM_005266249, XM_005266250, XM_011534903, XM_011534904, XM_011534905, XM_011534906 | AKAP11 | A kinase (PRKA) anchor protein 11 | 0.30 | 6.88 | -0.20 | 6.88 | pre |
| 11721753_a_at | NA | NM_001039210, NM_001099922, NM_001168385, NM_001257230, NM_001257231, NM_001257234, NM_001257235, NM_001257237, NM_001257239, NM_001257240, NM_001257241, NM_018466, NR_033125, XM_005262191, XM_006724693, XM_006724695, XM_006724697, XM_006724698, XM_011531028, XM_011531029, XM_011531030, XM_011531031, XM_011531032, XM_011531033, XM_011531034, XM_011531035, XM_011531036, XM_011531037, XR_938409 | ALG13 | ALG13, UDP-N-acetylglucosaminyltransferase subunit | 0.48 | 8.73 | 0.43 | 8.73 | pre |
| 11721754_x_at | NA | NM_001039210, NM_001099922, NM_001168385, NM_001257230, NM_001257231, NM_001257234, NM_001257235, NM_001257237, NM_001257239, NM_001257240, NM_001257241, NM_018466, NR_033125, XM_005262191, XM_006724693, XM_006724695, XM_006724697, XM_006724698, XM_011531028, XM_011531029, XM_011531030, XM_011531031, XM_011531032, XM_011531033, XM_011531034, XM_011531035, XM_011531036, XM_011531037, XR_938409 | ALG13 | ALG13, UDP-N-acetylglucosaminyltransferase subunit | 0.48 | 8.82 | 0.42 | 8.82 | pre |
| 11725114_x_at | NA | NM_001197030, NM_017747, NM_017978, NM_024668 | ANKHD1 | Ankyrin repeat and KH domain containing 1 | 0.35 | 8.28 | 0.28 | 8.28 | pre |
| 11757688_a_at | NA | NM_001197030, NM_017747, NM_017978, NM_024668 | ANKHD1 | Ankyrin repeat and KH domain containing 1 | 0.39 | 7.35 | 0.33 | 7.35 | pre |
| 11759780_at | ENST00000370944.4 | NM_030816, XM_005271234, XM_005271235, XM_006710929, XM_011542215, XR_946767 | ANKRD13C | Ankyrin repeat domain 13C | 0.56 | 8.03 | 0.80 | 8.03 | pre |
| 11724570_at | ENST00000083182.3 | NM_001282476, NM_006380, XM_011524198, XM_011524199 | APPBP2 | Amyloid beta precursor protein (cytoplasmic tail) binding protein 2 | 0.32 | 7.66 | 0.29 | 7.66 | pre |
| 11724573_at | ENST00000083182.3 | NM_001282476, NM_006380, XM_011524198, XM_011524199 | APPBP2 | Amyloid beta precursor protein (cytoplasmic tail) binding protein 2 | 0.35 | 6.84 | 0.25 | 6.84 | pre |
| 11761385_a_at | ENST00000400198.3 | NM_018011, XR_243043, XR_429268, XR_429269 | ARGLU1 | Arginine and glutamate rich 1 | 0.56 | 4.99 | 0.43 | 4.99 | pre |
| 11735743_at | ENST00000320767.2 | NM_025047 | ARL14 | ADP-ribosylation factor like gtpase 14 | 0.57 | 11.04 | 0.89 | 11.04 | pre |
| 11733140_s_at | ENST00000396663.1 | NM_001037164, NM_001195396, NM_005738, NM_212460 | ARL4A | ADP-ribosylation factor like gtpase 4A | 0.29 | 10.06 | 0.35 | 10.06 | pre |
| 11739230_a_at | ENST00000396663.1 | NM_001037164, NM_001195396, NM_005738, NM_212460 | ARL4A | ADP-ribosylation factor like gtpase 4A | 0.37 | 8.97 | 0.37 | 8.97 | pre |
| 11756387_x_at | ENST00000396663.1 | NM_001037164, NM_001195396, NM_005738, NM_212460 | ARL4A | ADP-ribosylation factor like gtpase 4A | 0.23 | 9.49 | 0.36 | 9.49 | pre |
| 11753615_a_at | ENST00000419534.2 | NM_018184, XM_005265295 | ARL8B | ADP-ribosylation factor like gtpase 8B | 0.35 | 9.69 | 0.13 | 9.69 | pre |
| 11753616_s_at | ENST00000419534.2 | NM_018184, XM_005265295 | ARL8B | ADP-ribosylation factor like gtpase 8B | 0.36 | 9.33 | 0.12 | 9.33 | pre |
| 11752314_a_at | ENST00000261168.4 | NM_001286514, NM_001286515, NM_018179, NM_181352, XM_005253424, XM_006719108, XM_006719109, XM_011520754, XM_011520755, XM_011520756, XM_011520757, XM_011520758, XM_011520759 | ATF7IP | Activating transcription factor 7 interacting protein | 0.34 | 7.42 | 0.09 | 7.42 | pre |
| 11726703_a_at | ENST00000370580.1 | NM_003921, XM_005271311, XM_011542397, XM_011542398, XM_011542399 | BCL10 | B-cell CLL/lymphoma 10 | 0.31 | 9.20 | 0.15 | 9.20 | pre |
| 11753497_a_at | ENST00000370580.1 | NM_003921, XM_005271311, XM_011542397, XM_011542398, XM_011542399 | BCL10 | B-cell CLL/lymphoma 10 | 0.33 | 7.93 | 0.28 | 7.93 | pre |
| 11759514_at | ENST00000376062.2 | NM_001191, NM_138578, XM_005260486, XM_005260487, XM_011528960, XM_011528961, XM_011528962, XM_011528963, XM_011528964, XM_011528965, XM_011528966, XR_936599 | BCL2L1 | BCL2-like 1 | 0.34 | 6.92 | 0.14 | 6.92 | pre |
| 11760008_at | ENST00000301633.4 | NM_001012270, NM_001012271, NM_001168, XR_243654, XR_934452 | BIRC5 | Baculoviral IAP repeat containing 5 | 0.31 | 4.43 | 0.14 | 4.43 | pre |
| 11736591_at | ENST00000040738.5 | NM_148894, XM_005248150, XM_005248151, XM_006713958, XM_011513827, XM_011513828, XM_011513829, XM_011513830 | BOD1L1 | Biorientation of chromosomes in cell division 1-like 1 | 0.41 | 8.10 | 0.01 | 8.10 | pre |
| 11743990_at | ENST00000263377.2 | NM_014299, NM_058243, XM_011527854, XM_011527855, XM_011527856 | BRD4 | Bromodomain containing 4 | 0.35 | 7.34 | 0.32 | 7.34 | pre |
| 11733023_s_at | ENST00000256015.3 | NM_001731 | BTG1 | B-cell translocation gene 1, anti-proliferative | 0.49 | 11.32 | 0.33 | 11.32 | pre |
| 11733024_x_at | ENST00000256015.3 | NM_001731 | BTG1 | B-cell translocation gene 1, anti-proliferative | 0.58 | 10.57 | 0.35 | 10.57 | pre |
| 11738248_a_at | ENST00000325636.4 | NM_001271562, NM_019021 | C11orf71 | Chromosome 11 open reading frame 71 | 0.98 | 6.95 | 0.83 | 6.95 | pre |
| 11759623_at | ENST00000325636.4 | NM_001271562, NM_019021 | C11orf71 | Chromosome 11 open reading frame 71 | 1.22 | 6.95 | 1.41 | 6.95 | pre |
| 11754802_s_at | ENST00000546651.2 | NM_207435, XM_005253882, XM_011538344, XM_011538345, XR_944547 | C12orf76 | Chromosome 12 open reading frame 76 | 0.38 | 5.72 | 0.40 | 5.72 | pre |
| 11743154_at | ENST00000327827.7 | NM_014117, XM_011522462, XR_932824 | C16orf72 | Chromosome 16 open reading frame 72 | 0.25 | 8.02 | 0.34 | 8.02 | pre |
| 11744385_a_at | ENST00000442039.2 | NM_001010878, NM_001272051 | C16orf91 | Chromosome 16 open reading frame 91 | 0.32 | 9.11 | 0.33 | 9.11 | pre |
| 11720619_a_at | ENST00000436106.2 | NM_152482, XM_005259506, XM_006722653 | C19orf25 | Chromosome 19 open reading frame 25 | 0.29 | 7.96 | 0.39 | 7.96 | pre |
| 11748882_a_at | ENST00000372525.5 | NM_024097, NR_040733 | C1orf50 | Chromosome 1 open reading frame 50 | 0.39 | 7.98 | 0.31 | 7.98 | pre |
| 11735041_a_at | NA | NM_198077, NR_024113 | C1orf52 | Chromosome 1 open reading frame 52 | 0.40 | 5.60 | 0.40 | 5.60 | pre |
| 11736981_a_at | ENST00000359558.2 | NM_001164737, NM_001164738, NM_001742 | CALCR | Calcitonin receptor | 0.31 | 3.63 | -0.08 | 3.63 | pre |
| 11739442_s_at | ENST00000523505.1 | NM_175884 | CCDC71L | Coiled-coil domain containing 71-like | 0.40 | 5.87 | 0.45 | 5.87 | pre |
| 11750185_a_at | ENST00000523505.1 | NM_175884 | CCDC71L | Coiled-coil domain containing 71-like | 0.31 | 9.13 | 0.33 | 9.13 | pre |
| 11715675_a_at | ENST00000340828.2 | NM_004060, NM_199246, XM_011534685, XR_941118 | CCNG1 | Cyclin G1 | 0.27 | 8.52 | 0.33 | 8.52 | pre |
| 11749306_a_at | ENST00000340828.2 | NM_004060, NM_199246, XM_011534685, XR_941118 | CCNG1 | Cyclin G1 | 0.27 | 7.59 | 0.42 | 7.59 | pre |
| 11729153_at | ENST00000199764.6 | NM_002483, XM_011526990 | CEACAM6 | Carcinoembryonic antigen-related cell adhesion molecule 6 (non-specific cross reacting antigen) | 0.43 | 9.82 | 0.03 | 9.82 | pre |
| 11736054_a_at | ENST00000395290.2 | NM_001025596, NM_001172639, NM_001172640, NM_006560, NM_198700, XM_011519847, XM_011519848, XM_011519849, XM_011519850, XM_011519851, XM_011519852, XM_011519853, XM_011519854, XM_011519855, XM_011519856, XM_011519857, XM_011519858, XM_011519859 | CELF1 | CUGBP, Elav-like family member 1 | 0.44 | 8.38 | 0.33 | 8.38 | pre |
| 11742899_a_at | ENST00000309534.6 | NM_001008390, NM_001195308, NM_003663, XM_011534170 | CGGBP1 | CGG triplet repeat binding protein 1 | 0.39 | 10.11 | 0.26 | 10.11 | pre |
| 11731477_at | ENST00000526991.2 | NM_020412 | CHMP1B | Charged multivesicular body protein 1B | 0.43 | 9.77 | 0.25 | 9.77 | pre |
| 11724747_a_at | ENST00000263780.4 | NM_001244644, NM_014043, XM_011533576 | CHMP2B | Charged multivesicular body protein 2B | 0.32 | 10.44 | 0.02 | 10.44 | pre |
| 11742061_a_at | ENST00000320585.6 | NM_001144073, NM_012124, XM_011542747, XM_011542748, XM_011542749 | CHORDC1 | Cysteine and histidine rich domain containing 1 | 0.47 | 6.54 | 0.22 | 6.54 | pre |
| 11743052_a_at | ENST00000320585.6 | NM_001144073, NM_012124, XM_011542747, XM_011542748, XM_011542749 | CHORDC1 | Cysteine and histidine rich domain containing 1 | 0.39 | 7.02 | 0.11 | 7.02 | pre |
| 11743053_a_at | ENST00000320585.6 | NM_001144073, NM_012124, XM_011542747, XM_011542748, XM_011542749 | CHORDC1 | Cysteine and histidine rich domain containing 1 | 0.46 | 6.91 | 0.20 | 6.91 | pre |
| 11748024_a_at | ENST00000316308.4 | NM_020666, XM_005265947, XM_005265948, XM_005265949, XM_006714895, XM_006714896, XM_011534601, XM_011534602, XM_011534603, XR_427807 | CLK4 | CDC like kinase 4 | 0.56 | 6.38 | 0.52 | 6.38 | pre |
| 11756853_a_at | ENST00000316308.4 | NM_020666, XM_005265947, XM_005265948, XM_005265949, XM_006714895, XM_006714896, XM_011534601, XM_011534602, XM_011534603, XR_427807 | CLK4 | CDC like kinase 4 | 0.37 | 7.18 | 0.33 | 7.18 | pre |
| 11745418_a_at | ENST00000349995.5 | NM_001204476, NM_031431, XM_011535266, XM_011535267, XM_011535268, XR_245400, XR_429222 | COG3 | Component of oligomeric golgi complex 3 | 0.57 | 5.01 | 0.52 | 5.01 | pre |
| 11724191_a_at | ENST00000388901.5 | NM_001143887, NM_004236 | COPS2 | COP9 signalosome subunit 2 | 0.31 | 9.31 | 0.25 | 9.31 | pre |
| 11724950_a_at | ENST00000538197.1 | NM_001177381, NM_001177382, NM_001177383, NM_001177384, NM_182485, NM_182646, XM_005248135, XM_011513777, XM_011513778, XM_011513779 | CPEB2 | Cytoplasmic polyadenylation element binding protein 2 | 0.55 | 5.78 | 0.40 | 5.78 | pre |
| 11724951_s_at | ENST00000538197.1 | NM_001177381, NM_001177382, NM_001177383, NM_001177384, NM_182485, NM_182646, XM_005248135, XM_011513777, XM_011513778, XM_011513779 | CPEB2 | Cytoplasmic polyadenylation element binding protein 2 | 0.35 | 7.37 | 0.40 | 7.37 | pre |
| 11719103_at | ENST00000198765.4 | NM_003909, XM_005251093 | CPNE3 | Copine III | 0.45 | 9.49 | -0.06 | 9.49 | pre |
| 11759649_x_at | ENST00000330387.6 | NM_001253775, NM_194071, XM_005250541, XM_011516492 | CREB3L2 | Camp responsive element binding protein 3-like 2 | 0.39 | 5.65 | 0.03 | 5.65 | pre |
| 11727223_a_at | ENST00000454498.2 | NM_001130518, NM_018371, NR_024040, XM_006716358, XM_006716359, XM_006716360, XM_006716361, XM_006716362, XM_006716363, XM_006716364, XM_011544577, XM_011544578, XM_011544579, XM_011544580, XM_011544581, XM_011544582, XM_011544583, XM_011544584, XM_011544585, XR_949441 | CSGALNACT1 | Chondroitin sulfate N-acetylgalactosaminyltransferase 1 | 0.34 | 5.15 | -0.04 | 5.15 | pre |
| 11732526_s_at | ENST00000454498.2 | NM_001130518, NM_018371, NR_024040, XM_006716358, XM_006716359, XM_006716360, XM_006716361, XM_006716362, XM_006716363, XM_006716364, XM_011544577, XM_011544578, XM_011544579, XM_011544580, XM_011544581, XM_011544582, XM_011544583, XM_011544584, XM_011544585, XR_949441 | CSGALNACT1 | Chondroitin sulfate N-acetylgalactosaminyltransferase 1 | 0.37 | 4.33 | -0.06 | 4.33 | pre |
| 11730863_at | ENST00000271277.6 | NM_018704, XM_011541781 | CTTNBP2NL | CTTNBP2 N-terminal like | 0.20 | 6.86 | 0.30 | 6.86 | pre |
| 11736519_x_at | ENST00000378426.1 | NM_144970, XM_005272589, XM_006724527, XM_006724528, XM_011543891 | CXorf38 | Chromosome X open reading frame 38 | 0.34 | 8.33 | 0.32 | 8.33 | pre |
| 11756170_a_at | ENST00000378426.1 | NM_144970, XM_005272589, XM_006724527, XM_006724528, XM_011543891 | CXorf38 | Chromosome X open reading frame 38 | 0.42 | 7.65 | 0.44 | 7.65 | pre |
| 11747104_s_at | ENST00000260630.3 | NM_000104 | CYP1B1 | Cytochrome P450, family 1, subfamily B, polypeptide 1 | 0.62 | 7.10 | 0.63 | 7.10 | pre |
| 11733379_at | ENST00000333141.5 | NM_001029955 | DCAF4L1 | DDB1 and CUL4 associated factor 4-like 1 | 0.43 | 3.44 | -0.11 | 3.44 | pre |
| 11729626_a_at | ENST00000310827.4 | NM_001003725, NM_005828, NR_073585 | DCAF7 | DDB1 and CUL4 associated factor 7 | 0.30 | 8.30 | 0.11 | 8.30 | pre |
| 11745757_a_at | ENST00000379892.4 | NM_001195415, NM_001195416, NM_001195430, NM_004734, XM_005266592, XM_006719893 | DCLK1 | Doublecortin-like kinase 1 | -0.42 | 3.94 | 0.32 | 3.94 | pre |
| 11760188_x_at | ENST00000215770.5 | NM_001084393, XM_011529816, XM_011546905 | DDTL | D-dopachrome tautomerase-like | 0.59 | 5.23 | 0.33 | 5.23 | pre |
| 11761623_at | ENST00000215770.5 | NM_001084393, XM_011529816, XM_011546905 | DDTL | D-dopachrome tautomerase-like | 0.38 | 5.57 | 0.14 | 5.57 | pre |
| 11721580_a_at | ENST00000438257.4 | NM_000793, NM_001007023, NM_001242502, NM_001242503, NM_013989 | DIO2 | Deiodinase, iodothyronine, type II | 0.96 | 6.87 | 1.10 | 6.87 | pre |
| 11741639_a_at | ENST00000438257.4 | NM_000793, NM_001007023, NM_001242502, NM_001242503, NM_013989 | DIO2 | Deiodinase, iodothyronine, type II | 0.57 | 6.28 | 0.99 | 6.28 | pre |
| 11749826_a_at | ENST00000438257.4 | NM_000793, NM_001007023, NM_001242502, NM_001242503, NM_013989 | DIO2 | Deiodinase, iodothyronine, type II | 0.78 | 5.48 | 1.07 | 5.48 | pre |
| 11749976_a_at | ENST00000452085.3 | NM_001080976, NM_013352, XM_011535785 | DSE | Dermatan sulfate epimerase | 0.39 | 6.61 | 0.33 | 6.61 | pre |
| 11733776_a_at | ENST00000226004.3 | NM_004090 | DUSP3 | Dual specificity phosphatase 3 | 0.33 | 7.81 | 0.11 | 7.81 | pre |
| 11748476_a_at | ENST00000226004.3 | NM_004090 | DUSP3 | Dual specificity phosphatase 3 | 0.35 | 8.04 | 0.10 | 8.04 | pre |
| 11753617_a_at | ENST00000226004.3 | NM_004090 | DUSP3 | Dual specificity phosphatase 3 | 0.33 | 7.80 | 0.11 | 7.80 | pre |
| 11722049_a_at | ENST00000279488.7 | NM_001946, NM_022652 | DUSP6 | Dual specificity phosphatase 6 | 0.31 | 9.37 | 0.20 | 9.37 | pre |
| 11741980_a_at | ENST00000279488.7 | NM_001946, NM_022652 | DUSP6 | Dual specificity phosphatase 6 | 0.31 | 9.87 | 0.13 | 9.87 | pre |
| 11738815_at | NA | NM_005228, NM_201282, NM_201283, NM_201284 | EGFR | Epidermal growth factor receptor | 0.32 | 3.75 | 0.09 | 3.75 | pre |
| 11762479_at | NA | NM_005228, NM_201282, NM_201283, NM_201284 | EGFR | Epidermal growth factor receptor | 0.38 | 3.87 | 0.13 | 3.87 | pre |
| 11730510_at | ENST00000326282.4 | NM_152361 | EID2B | EP300 interacting inhibitor of differentiation 2B | 0.43 | 5.57 | 0.44 | 5.57 | pre |
| 11724724_a_at | ENST00000216554.3 | NM_001969, NM_183004 | EIF5 | Eukaryotic translation initiation factor 5 | 0.31 | 11.45 | 0.22 | 11.45 | pre |
| 11758020_s_at | ENST00000216554.3 | NM_001969, NM_183004 | EIF5 | Eukaryotic translation initiation factor 5 | 0.31 | 11.01 | 0.35 | 11.01 | pre |
| 11736478_a_at | ENST00000237853.4 | NM_012081, XM_006714575, XM_011543280 | ELL2 | Elongation factor, RNA polymerase II, 2 | 0.55 | 6.67 | 0.32 | 6.67 | pre |
| 11749208_a_at | ENST00000237853.4 | NM_012081, XM_006714575, XM_011543280 | ELL2 | Elongation factor, RNA polymerase II, 2 | 0.33 | 6.39 | 0.37 | 6.39 | pre |
| 11752840_a_at | ENST00000237853.4 | NM_012081, XM_006714575, XM_011543280 | ELL2 | Elongation factor, RNA polymerase II, 2 | 0.33 | 6.73 | 0.27 | 6.73 | pre |
| 11725354_at | ENST00000322716.5 | NM_014805 | EPM2AIP1 | EPM2A (laforin) interacting protein 1 | 0.33 | 5.13 | 0.24 | 5.13 | pre |
| 11735259_a_at | ENST00000276461.5 | NM_001003790, NM_001003791, NM_007175, XM_005273392, XM_006716280 | ERLIN2 | ER lipid raft associated 2 | 0.38 | 5.07 | 0.03 | 5.07 | pre |
| 11741586_a_at | ENST00000276461.5 | NM_001003790, NM_001003791, NM_007175, XM_005273392, XM_006716280 | ERLIN2 | ER lipid raft associated 2 | 0.38 | 7.07 | 0.18 | 7.07 | pre |
| 11741587_x_at | ENST00000276461.5 | NM_001003790, NM_001003791, NM_007175, XM_005273392, XM_006716280 | ERLIN2 | ER lipid raft associated 2 | 0.30 | 6.85 | 0.05 | 6.85 | pre |
| 11745025_a_at | ENST00000276461.5 | NM_001003790, NM_001003791, NM_007175, XM_005273392, XM_006716280 | ERLIN2 | ER lipid raft associated 2 | 0.38 | 6.39 | 0.07 | 6.39 | pre |
| 11745026_a_at | ENST00000276461.5 | NM_001003790, NM_001003791, NM_007175, XM_005273392, XM_006716280 | ERLIN2 | ER lipid raft associated 2 | 0.34 | 6.06 | -0.06 | 6.06 | pre |
| 11725203_a_at | ENST00000269214.5 | NM_052911, XM_011525798, XM_011525799, XR_935191 | ESCO1 | Establishment of sister chromatid cohesion N-acetyltransferase 1 | 0.36 | 7.20 | 0.01 | 7.20 | pre |
| 11760883_at | ENST00000301838.4 | NM_003824 | FADD | Fas (TNFRSF6)-associated via death domain | 0.72 | 5.15 | 0.31 | 5.15 | pre |
| 11717406_a_at | ENST00000423591.1 | NM_198841, XM_005251736 | FAM120AOS | Family with sequence similarity 120A opposite strand | 0.31 | 5.66 | 0.37 | 5.66 | pre |
| 11728317_a_at | ENST00000394264.3 | NM_138333 | FAM122A | Family with sequence similarity 122A | 0.26 | 7.44 | 0.34 | 7.44 | pre |
| 11752722_a_at | ENST00000394264.3 | NM_138333 | FAM122A | Family with sequence similarity 122A | 0.35 | 5.56 | 0.12 | 5.56 | pre |
| 11739380_a_at | ENST00000585682.1 | NM_001031700, NM_001128424, NM_016613, XR_938744 | FAM198B | Family with sequence similarity 198, member B | 0.34 | 6.16 | 0.19 | 6.16 | pre |
| 11744152_a_at | ENST00000327858.6 | NM_001996, NM_006485, NM_006486, NM_006487 | FBLN1 | Fibulin 1 | 0.28 | 4.95 | 0.32 | 4.95 | pre |
| 11719394_a_at | ENST00000517956.1 | NM_001242463, NM_058229, NM_148177 | FBXO32 | F-box protein 32 | 0.71 | 7.61 | 0.35 | 7.61 | pre |
| 11759612_at | ENST00000336824.4 | NM_001135095, NM_022763, XM_011513083, XM_011513084 | FNDC3B | Fibronectin type III domain containing 3B | 0.45 | 8.13 | 0.60 | 8.13 | pre |
| 11744435_a_at | ENST00000279488.7 | NM_001946, NM_022652 | FRS2 | Dual specificity phosphatase 6 | 0.32 | 9.44 | 0.20 | 9.44 | pre |
| 11750929_a_at | ENST00000550389.1 | NM_001042555, NM_001278351, NM_001278353, NM_001278354, NM_001278355, NM_001278356, NM_001278357, NM_006654 | FRS2 | Fibroblast growth factor receptor substrate 2 | 0.30 | 5.69 | 0.28 | 5.69 | pre |
| 11761105_at | ENST00000370328.3 | NM_004961, NM_021984, NM_021987, NM_021990, XM_006724813, XM_011531135, XM_011531136, XM_011531137, XM_011531138, XM_011531139, XM_011531140 | GABRE | Gamma-aminobutyric acid (GABA) A receptor, epsilon | 0.35 | 3.44 | 0.26 | 3.44 | pre |
| 11753719_a_at | ENST00000358056.3 | NM_004124, XM_005267541, XM_011536660 | GMFB | Glia maturation factor, beta | 0.38 | 7.35 | 0.22 | 7.35 | pre |
| 11753720_x_at | ENST00000358056.3 | NM_004124, XM_005267541, XM_011536660 | GMFB | Glia maturation factor, beta | 0.37 | 7.77 | 0.16 | 7.77 | pre |
| 11717682_a_at | ENST00000439174.2 | NM_001282425, NM_006572, XM_011524202, XR_934352 | GNA13 | Guanine nucleotide binding protein (G protein), alpha 13 | 0.42 | 5.01 | 0.53 | 5.01 | pre |
| 11748689_a_at | ENST00000439174.2 | NM_001282425, NM_006572, XM_011524202, XR_934352 | GNA13 | Guanine nucleotide binding protein (G protein), alpha 13 | 0.28 | 6.33 | 0.32 | 6.33 | pre |
| 11715721_a_at | ENST00000388712.3 | NM_001099268, NM_016548, NM_177937 | GOLM1 | Golgi membrane protein 1 | 0.33 | 7.96 | 0.19 | 7.96 | pre |
| 11724463_a_at | NA | NM_001005340, NM_002510, XM_005249578 | GPNMB | Glycoprotein (transmembrane) nmb | 0.82 | 4.68 | 0.57 | 4.68 | pre |
| 11752934_a_at | ENST00000251808.3 | NM_024915, XM_011517305, XM_011517306, XM_011517307 | GRHL2 | Grainyhead-like transcription factor 2 | 0.38 | 5.75 | 0.16 | 5.75 | pre |
| 11760785_s_at | ENST00000392870.2 | NM_005308, XM_005269707, XM_005269708, XM_011539699 | GRK5 | G protein-coupled receptor kinase 5 | 0.36 | 4.10 | -0.07 | 4.10 | pre |
| 11748895_a_at | NA | NM_152407 | GRPEL2 | Grpe-like 2, mitochondrial (E. Coli) | 0.29 | 6.46 | 0.42 | 6.46 | pre |
| 11746129_a_at | ENST00000540225.1 | NM_000849, NR_024537, XM_011541296 | GSTM3 | Glutathione S-transferase mu 3 (brain) | 0.32 | 5.25 | -0.15 | 5.25 | pre |
| 11749079_a_at | ENST00000283875.5 | NM_005513, XM_011512744, XM_011512745 | GTF2E1 | General transcription factor IIE subunit 1 | 0.30 | 5.91 | 0.19 | 5.91 | pre |
| 11724160_a_at | ENST00000398675.3 | NM_001099650, NM_173601 | GXYLT1 | Glucoside xylosyltransferase 1 | 0.35 | 7.03 | 0.51 | 7.03 | pre |
| 11724161_a_at | ENST00000398675.3 | NM_001099650, NM_173601 | GXYLT1 | Glucoside xylosyltransferase 1 | 0.33 | 6.74 | 0.32 | 6.74 | pre |
| 11724545_at | ENST00000229330.4 | NM_013320, XM_011538247, XR_944526, XR_944527 | HCFC2 | Host cell factor C2 | 0.26 | 5.96 | 0.35 | 5.96 | pre |
| 11736244_s_at | ENST00000602637.1 | NM_003512 | HIST1H2AC | Histone cluster 1, h2ac | 0.37 | 7.64 | 0.44 | 7.64 | pre |
| 11734796_s_at | ENST00000244601.3 | NM_003518 | HIST1H2BG | Histone cluster 1, h2bg | 1.01 | 7.36 | 0.47 | 7.36 | pre |
| 11734797_x_at | ENST00000244601.3 | NM_003518 | HIST1H2BG | Histone cluster 1, h2bg | 1.20 | 6.83 | 0.59 | 6.83 | pre |
| 11759111_x_at | ENST00000356350.2 | NM_003524 | HIST1H2BH | Histone cluster 1, h2bh | 0.65 | 6.41 | 1.06 | 6.41 | pre |
| 11732032_a_at | ENST00000403681.2 | NM_001015886, NM_001300918, NM_001300919, NM_003483, NM_003484 | HMGA2 | High mobility group AT-hook 2 | 0.81 | 4.92 | 0.16 | 4.92 | pre |
| 11762061_at | ENST00000403681.2 | NM_001015886, NM_001300918, NM_001300919, NM_003483, NM_003484 | HMGA2 | High mobility group AT-hook 2 | 0.38 | 3.24 | 0.05 | 3.24 | pre |
| 11762062_x_at | ENST00000403681.2 | NM_001015886, NM_001300918, NM_001300919, NM_003483, NM_003484 | HMGA2 | High mobility group AT-hook 2 | 0.42 | 3.41 | 0.07 | 3.41 | pre |
| 11759794_at | ENST00000278353.4 | NM_016142, XM_011520156 | HSD17B12 | Hydroxysteroid (17-beta) dehydrogenase 12 | 0.53 | 5.56 | 0.32 | 5.56 | pre |
| 11749913_a_at | ENST00000285667.3 | NM_006948 | HSPA13 | Heat shock protein 70kda family, member 13 | 0.34 | 7.29 | 0.27 | 7.29 | pre |
| 11760820_at | ENST00000340855.6 | NM_000202, NM_001166550, NM_006123, NR_104128 | IDS | Iduronate 2-sulfatase | 0.65 | 3.96 | 0.10 | 3.96 | pre |
| 11760820_at | ENST00000422081.2 | NM_000202, NM_001166550, NM_006123, NR_104128 | IDS | Iduronate 2-sulfatase | 0.65 | 3.96 | 0.10 | 3.96 | pre |
| 11746463_a_at | NA | NM_000600, XM_005249745, XM_011515390, XM_011515391 | IL6 | Interleukin 6 | 0.73 | 9.79 | 0.99 | 9.79 | pre |
| 11736509_x_at | ENST00000344086.4 | NM_000565, NM_001206866, NM_181359, XM_005245139, XM_005245140, XM_006711298, XM_006711299 | IL6R | Interleukin 6 receptor | 0.33 | 4.43 | 0.02 | 4.43 | pre |
| 11741959_x_at | ENST00000344086.4 | NM_000565, NM_001206866, NM_181359, XM_005245139, XM_005245140, XM_006711298, XM_006711299 | IL6R | Interleukin 6 receptor | 0.54 | 4.87 | 0.23 | 4.87 | pre |
| 11730758_a_at | ENST00000381287.4 | NM_001190981, NM_002184, NM_175767, NR_120480, XM_011543376, XM_011543377 | IL6ST | Interleukin 6 signal transducer | 0.39 | 6.20 | 0.16 | 6.20 | pre |
| 11734299_at | ENST00000311234.4 | NM_001039937, NM_001039938, NM_001306091, NM_012141, XM_005266340, XM_005266341, XM_011535040, XM_011535041 | INTS6 | Integrator complex subunit 6 | 0.43 | 5.66 | 0.78 | 5.66 | pre |
| 11751761_a_at | ENST00000311234.4 | NM_001039937, NM_001039938, NM_001306091, NM_012141, XM_005266340, XM_005266341, XM_011535040, XM_011535041 | INTS6 | Integrator complex subunit 6 | 0.30 | 6.54 | 0.13 | 6.54 | pre |
| 11751762_s_at | ENST00000311234.4 | NM_001039937, NM_001039938, NM_001306091, NM_012141, XM_005266340, XM_005266341, XM_011535040, XM_011535041 | INTS6 | Integrator complex subunit 6 | 0.21 | 5.75 | 0.45 | 5.75 | pre |
| 11747473_a_at | ENST00000222573.4 | NM_002214, XM_011515392, XM_011515393, XM_011515394, XM_011515395, XM_011515396 | ITGB8 | Integrin beta 8 | 0.31 | 6.62 | 0.12 | 6.62 | pre |
| 11749103_a_at | ENST00000222573.4 | NM_002214, XM_011515392, XM_011515393, XM_011515394, XM_011515395, XM_011515396 | ITGB8 | Integrin beta 8 | 0.36 | 7.01 | 0.22 | 7.01 | pre |
| 11751989_a_at | ENST00000222573.4 | NM_002214, XM_011515392, XM_011515393, XM_011515394, XM_011515395, XM_011515396 | ITGB8 | Integrin beta 8 | 0.33 | 5.06 | 0.06 | 5.06 | pre |
| 11740266_at | ENST00000458427.1 | NM_001261833, NM_003772 | JRKL | JRK-like | 0.21 | 7.07 | 0.32 | 7.07 | pre |
| 11749733_a_at | ENST00000458427.1 | NM_001261833, NM_003772 | JRKL | JRK-like | 0.21 | 5.55 | 0.36 | 5.55 | pre |
| 11749734_s_at | ENST00000458427.1 | NM_001261833, NM_003772 | JRKL | JRK-like | 0.25 | 6.60 | 0.61 | 6.60 | pre |
| 11752031_a_at | ENST00000361413.3 | NM_014804, XM_006721611, XM_006721612, XM_011524090, XM_011524091, XM_011524092, XM_011524093, XM_011524094, XM_011524095, XM_011524096, XM_011524097, XM_011524098, XM_011524099, XM_011524100, XR_934126 | KIAA0753 | Kiaa0753 | 0.26 | 6.03 | 0.32 | 6.03 | pre |
| 11744572_a_at | ENST00000377687.4 | NM_001286818, NM_001730 | KLF5 | Kruppel-like factor 5 (intestinal) | 0.40 | 10.34 | 0.21 | 10.34 | pre |
| 11722282_a_at | ENST00000230538.7 | NM_001105206, NM_001105207, NM_001105208, NM_001105209, NM_002290, XM_005266983, XM_005266984, XM_011535821 | LAMA4 | Laminin, alpha 4 | 0.56 | 7.21 | 0.09 | 7.21 | pre |
| 11763484_x_at | ENST00000359591.4 | NM_014045, XM_005267510 | LRP10 | LDL receptor related protein 10 | 0.37 | 5.81 | 0.15 | 5.81 | pre |
| 11739317_at | ENST00000409947.1 | NM_006609 | MAP3K2 | Mitogen-activated protein kinase kinase kinase 2 | 0.31 | 7.42 | 0.19 | 7.42 | pre |
| 11754144_a_at | ENST00000397752.3 | NM_000245, NM_001127500, XM_006715990, XM_006715991, XM_011516223 | MET | MET proto-oncogene, receptor tyrosine kinase | 0.56 | 5.92 | 0.25 | 5.92 | pre |
| 11724498_a_at | ENST00000416983.3 | NM_001166343, NM_002405, NR_029413 | MFNG | MFNG O-fucosylpeptide 3-beta-N-acetylglucosaminyltransferase | 0.24 | 4.55 | 0.41 | 4.55 | pre |
| 11724499_at | ENST00000416983.3 | NM_001166343, NM_002405, NR_029413 | MFNG | MFNG O-fucosylpeptide 3-beta-N-acetylglucosaminyltransferase | 0.31 | 5.97 | 0.02 | 5.97 | pre |
| 11747759_a_at | ENST00000370783.3 | NM_001306188, NM_019556, XM_005262446, XM_011531366 | MOSPD1 | Motile sperm domain containing 1 | 0.35 | 7.17 | 0.11 | 7.17 | pre |
| 11717998_a_at | ENST00000306984.6 | NM_138701 | MPLKIP | M-phase specific PLK1 interacting protein | 0.43 | 10.44 | 0.24 | 10.44 | pre |
| 11717999_at | ENST00000306984.6 | NM_138701 | MPLKIP | M-phase specific PLK1 interacting protein | 0.45 | 11.00 | 0.26 | 11.00 | pre |
| 11718000_x_at | ENST00000306984.6 | NM_138701 | MPLKIP | M-phase specific PLK1 interacting protein | 0.42 | 10.70 | 0.25 | 10.70 | pre |
| 11725634_at | ENST00000253686.2 | NM_022497, XM_011534014, XR_940490, XR_940491, XR_940492 | MRPS25 | Mitochondrial ribosomal protein S25 | 0.34 | 6.68 | 0.32 | 6.68 | pre |
| 11760918_a_at | ENST00000344641.3 | NM_001173512, NM_138777, NM_199176, NM_199177, XM_005252302, XM_011519183, XM_011519184, XM_011519185, XM_011519186, XM_011519187, XR_242607, XR_428543 | MRRF | Mitochondrial ribosome recycling factor | 0.25 | 4.65 | 0.39 | 4.65 | pre |
| 11722271_at | ENST00000301919.4 | NM_032424, XM_005271697, XM_005271698, XM_005271699, XM_005271701, XM_011543022 | MSANTD4 | Myb/SANT-like DNA-binding domain containing 4 with coiled-coils | 0.33 | 6.38 | 0.41 | 6.38 | pre |
| 11736379_at | ENST00000309993.2 | NM_001145417, NM_018133, XM_005247571, XM_005247572, XM_006713684, XM_011512949 | MSL2 | Male-specific lethal 2 homolog (Drosophila) | 0.24 | 6.67 | 0.32 | 6.67 | pre |
| 11726725_a_at | ENST00000410026.2 | NM_001031716, NM_001254736, NM_022837, NR_045622, NR_045623, XR_922988 | NABP1 | Nucleic acid binding protein 1 | 0.81 | 9.49 | 0.75 | 9.49 | pre |
| 11726726_s_at | ENST00000410026.2 | NM_001031716, NM_001254736, NM_022837, NR_045622, NR_045623, XR_922988 | NABP1 | Nucleic acid binding protein 1 | 0.76 | 9.55 | 0.71 | 9.55 | pre |
| 11726727_a_at | ENST00000410026.2 | NM_001031716, NM_001254736, NM_022837, NR_045622, NR_045623, XR_922988 | NABP1 | Nucleic acid binding protein 1 | 0.72 | 8.61 | 0.75 | 8.61 | pre |
| 11726728_a_at | ENST00000410026.2 | NM_001031716, NM_001254736, NM_022837, NR_045622, NR_045623, XR_922988 | NABP1 | Nucleic acid binding protein 1 | 0.50 | 5.73 | 0.48 | 5.73 | pre |
| 11730320_a_at | ENST00000379446.5 | NM_001142393, NM_001271033, NM_006403, NM_182966, NR_073131 | NEDD9 | Neural precursor cell expressed, developmentally down-regulated 9 | 0.67 | 6.44 | 0.41 | 6.44 | pre |
| 11763503_a_at | ENST00000358273.4 | NM_000267, NM_001042492, NM_001128147, XM_005257983, XM_005257984, XM_006721922, XM_006721923, XM_006721924, XM_006721925, XM_006721926, XM_006721927, XM_006721928, XM_011524852, XM_011524853, XM_011524854, XM_011524855, XM_011524856, XM_011524857 | NF1 | Neurofibromin 1 | 0.40 | 5.07 | 0.16 | 5.07 | pre |
| 11745631_a_at | ENST00000380060.3 | NM_001136024, NM_001291867, NM_001291868, NM_198270, XM_011545528 | NHS | Nance-Horan syndrome (congenital cataracts and dental anomalies) | 0.38 | 5.52 | 0.02 | 5.52 | pre |
| 11743137_a_at | ENST00000366595.3 | NM_002508, XM_011544195 | NID1 | Nidogen 1 | 0.35 | 5.22 | 0.05 | 5.22 | pre |
| 11719188_at | ENST00000369535.4 | NM_002524 | NRAS | Neuroblastoma RAS viral (v-ras) oncogene homolog | 0.22 | 8.98 | 0.33 | 8.98 | pre |
| 11728215_a_at | ENST00000374875.1 | NM_001024628, NM_001024629, NM_001244972, NM_001244973, NM_003873, NR_045259, XM_006717521, XM_006717522, XM_006717523, XM_006717524, XM_006717525, XM_006717526, XM_011519755, XM_011519756 | NRP1 | Neuropilin 1 | 0.34 | 7.71 | 0.14 | 7.71 | pre |
| 11763489_a_at | ENST00000439151.2 | NM_022455, NM_172349, XM_005265959, XM_005265960, XM_005265961, XM_005265962, XM_011534610, XM_011534611, XM_011534612, XM_011534613, XM_011534614, XM_011534615, XM_011534616, XM_011534617 | NSD1 | Nuclear receptor binding SET domain protein 1 | 0.42 | 7.59 | 0.12 | 7.59 | pre |
| 11746950_a_at | ENST00000258742.5 | NM_007342, XM_005249592, XM_005249593, XM_011515104, XR_926913 | NUPL2 | Nucleoporin like 2 | 0.33 | 9.13 | 0.15 | 9.13 | pre |
| 11747896_a_at | ENST00000258742.5 | NM_007342, XM_005249592, XM_005249593, XM_011515104, XR_926913 | NUPL2 | Nucleoporin like 2 | 0.38 | 7.54 | 0.10 | 7.54 | pre |
| 11760676_at | ENST00000258742.5 | NM_007342, XM_005249592, XM_005249593, XM_011515104, XR_926913 | NUPL2 | Nucleoporin like 2 | 0.31 | 5.28 | 0.14 | 5.28 | pre |
| 11756310_a_at | ENST00000372106.1 | NM_001242617, NM_001242618, NM_018698, XM_011530989 | NXT2 | Nuclear transport factor 2-like export factor 2 | 0.32 | 6.96 | 0.29 | 6.96 | pre |
| 11746064_a_at | ENST00000373719.3 | NM_003605, NM_181672, NM_181673, XM_005262308 | OGT | O-linked N-acetylglucosamine (glcnac) transferase | 0.42 | 6.86 | 0.07 | 6.86 | pre |
| 11760246_at | ENST00000373719.3 | NM_003605, NM_181672, NM_181673, XM_005262308 | OGT | O-linked N-acetylglucosamine (glcnac) transferase | 0.94 | 4.42 | 0.35 | 4.42 | pre |
| 11723733_a_at | ENST00000285420.4 | NM_001286745, NM_016023, XM_011517129 | OTUD6B | OTU domain containing 6B | 0.26 | 6.68 | 0.37 | 6.68 | pre |
| 11719008_a_at | NA | NM_001252006, NM_001252007, NM_001293627, NM_001293628, NM_001293632, NM_032632, XM_005267281, XM_005267282 | PAPOLA | Poly(A) polymerase alpha | 0.33 | 10.70 | 0.20 | 10.70 | pre |
| 11744392_a_at | NA | NM_001252006, NM_001252007, NM_001293627, NM_001293628, NM_001293632, NM_032632, XM_005267281, XM_005267282 | PAPOLA | Poly(A) polymerase alpha | 0.33 | 10.68 | 0.24 | 10.68 | pre |
| 11744393_x_at | NA | NM_001252006, NM_001252007, NM_001293627, NM_001293628, NM_001293632, NM_032632, XM_005267281, XM_005267282 | PAPOLA | Poly(A) polymerase alpha | 0.34 | 11.31 | 0.26 | 11.31 | pre |
| 11727017_s_at | ENST00000328252.3 | NM_002581, XM_006717129 | PAPPA | Pregnancy-associated plasma protein A, pappalysin 1 | 0.31 | 5.68 | -0.05 | 5.68 | pre |
| 11754579_at | ENST00000328252.3 | NM_002581, XM_006717129 | PAPPA | Pregnancy-associated plasma protein A, pappalysin 1 | 0.35 | 6.10 | -0.11 | 6.10 | pre |
| 11753786_a_at | ENST00000371610.2 | NM_032521 | PARD6B | Par-6 family cell polarity regulator beta | 0.60 | 7.84 | 0.34 | 7.84 | pre |
| 11760870_at | ENST00000378128.3 | NM_017915, XM_011538506, XM_011538507, XM_011538508, XM_011538509, XM_011538510, XM_011538511, XM_011538512, XM_011538513, XM_011538514, XM_011538515, XM_011538516, XM_011538517, XM_011538518, XM_011538519, XM_011538520 | PARPBP | PARP1 binding protein | 0.41 | 3.84 | 0.25 | 3.84 | pre |
| 11720870_a_at | ENST00000401827.3 | NM_001242318, NM_002603, NM_002604, XM_011517540 | PDE7A | Phosphodiesterase 7A | 0.32 | 5.85 | 0.04 | 5.85 | pre |
| 11744571_a_at | NA | NM_020381, XM_011535956, XM_011535957, XM_011535958, XM_011535959, XM_011535960, XM_011535961, XM_011535962, XM_011535963 | PDSS2 | Prenyl (decaprenyl) diphosphate synthase, subunit 2 | 0.45 | 6.79 | 0.18 | 6.79 | pre |
| 11727890_a_at | ENST00000395131.1 | NM_001185181, NM_001265595, NM_001265596, NM_014260 | PFDN6 | Prefoldin subunit 6 | 0.36 | 9.11 | 0.10 | 9.11 | pre |
| 11747152_a_at | NA | NM_001134437, NM_001134438, NM_001134439, NM_145753 | PHLDB2 | Pleckstrin homology-like domain, family B, member 2 | 0.46 | 6.35 | 0.08 | 6.35 | pre |
| 11746259_a_at | ENST00000542278.1 | NM_002641, NM_020472, NM_020473, NR_033835, NR_033836, XM_011545539 | PIGA | Phosphatidylinositol glycan anchor biosynthesis class A | 0.37 | 6.41 | 0.30 | 6.41 | pre |
| 11746260_x_at | ENST00000542278.1 | NM_002641, NM_020472, NM_020473, NR_033835, NR_033836, XM_011545539 | PIGA | Phosphatidylinositol glycan anchor biosynthesis class A | 0.39 | 5.99 | 0.41 | 5.99 | pre |
| 11741174_s_at | ENST00000396418.2 | NM_006823, NM_181839 | PKIA | Protein kinase (camp-dependent, catalytic) inhibitor alpha | 0.31 | 7.45 | 0.11 | 7.45 | pre |
| 11754172_a_at | ENST00000396418.2 | NM_006823, NM_181839 | PKIA | Protein kinase (camp-dependent, catalytic) inhibitor alpha | 0.37 | 4.95 | 0.20 | 4.95 | pre |
| 11763261_x_at | ENST00000396418.2 | NM_006823, NM_181839 | PKIA | Protein kinase (camp-dependent, catalytic) inhibitor alpha | 0.55 | 5.53 | 0.25 | 5.53 | pre |
| 11759808_a_at | NA | NM_001193434, NM_001193435, NM_024889, NM_182601, XM_005270162, XM_005270163, XM_011540171, XM_011540172, XM_011540173, XM_011540174, XM_011540175, XM_011540176, XR_945813, XR_945814 | PLEKHS1 | Pleckstrin homology domain containing, family S member 1 | 0.36 | 3.97 | -0.24 | 3.97 | pre |
| 11759765_at | ENST00000342435.4 | NM_021105, XM_005247538, XM_011512904, XM_011512905, XM_011512906, XM_011512907 | PLSCR1 | Phospholipid scramblase 1 | 0.58 | 7.38 | 0.58 | 7.38 | pre |
| 11716988_s_at | ENST00000334351.7 | NM_017761 | PNRC2 | Proline-rich nuclear receptor coactivator 2 | 0.30 | 10.26 | 0.34 | 10.26 | pre |
| 11716989_s_at | ENST00000334351.7 | NM_017761 | PNRC2 | Proline-rich nuclear receptor coactivator 2 | 0.43 | 9.70 | 0.44 | 9.70 | pre |
| 11758304_s_at | ENST00000334351.7 | NM_017761 | PNRC2 | Proline-rich nuclear receptor coactivator 2 | 0.43 | 9.29 | 0.46 | 9.29 | pre |
| 11728759_a_at | NA | NM_001143787, NM_001143788, NM_001143789, NM_016488, NM_201438, NM_201439, NM_201440, NM_201515, XM_005268962, XM_005268965, XM_011538457, XM_011538458, XM_011538459, XM_011538460, XM_011538461, XM_011538462, XM_011538463, XM_011538464, XM_011538465, XM_011538466, XM_011538467, XM_011538468, XM_011538469, XM_011538470, XM_011538471, XM_011538472, XM_011538473, XM_011538474, XM_011538475 | PPHLN1 | Periphilin 1 | 0.33 | 6.84 | 0.38 | 6.84 | pre |
| 11752851_a_at | NA | NM_001143787, NM_001143788, NM_001143789, NM_016488, NM_201438, NM_201439, NM_201440, NM_201515, XM_005268962, XM_005268965, XM_011538457, XM_011538458, XM_011538459, XM_011538460, XM_011538461, XM_011538462, XM_011538463, XM_011538464, XM_011538465, XM_011538466, XM_011538467, XM_011538468, XM_011538469, XM_011538470, XM_011538471, XM_011538472, XM_011538473, XM_011538474, XM_011538475 | PPHLN1 | Periphilin 1 | 0.37 | 6.21 | 0.31 | 6.21 | pre |
| 11760036_at | ENST00000521072.2 | NM_001111298, NM_001286360, NM_001286361, NM_173672, NR_104429, XM_011535764, XM_011535765, XM_011535766, XM_011535767, XM_011535768, XM_011535769 | PPIL6 | Peptidylprolyl isomerase (cyclophilin)-like 6 | 0.35 | 5.45 | 0.27 | 5.45 | pre |
| 11763834_a_at | ENST00000409137.3 | NM_001080545, NM_001261424, NM_001261425, NR_048566, NR_048567 | PPP1R1C | Protein phosphatase 1, regulatory (inhibitor) subunit 1C | 0.31 | 6.69 | -0.10 | 6.69 | pre |
| 11717221_s_at | NA | NM_001142353, NM_001142354, NM_001289968, NM_001289969, NM_021132, XM_005269944, XM_011539921, XM_011539922, XM_011539923 | PPP3CB | Protein phosphatase 3, catalytic subunit, beta isozyme | 0.26 | 9.72 | 0.34 | 9.72 | pre |
| 11748282_a_at | NA | NM_001142353, NM_001142354, NM_001289968, NM_001289969, NM_021132, XM_005269944, XM_011539921, XM_011539922, XM_011539923 | PPP3CB | Protein phosphatase 3, catalytic subunit, beta isozyme | 0.30 | 8.11 | 0.37 | 8.11 | pre |
| 11736432_x_at | ENST00000356692.5 | NM_174907 | PPP4R2 | Protein phosphatase 4, regulatory subunit 2 | 0.40 | 8.25 | 0.22 | 8.25 | pre |
| 11759561_s_at | ENST00000356692.5 | NM_174907 | PPP4R2 | Protein phosphatase 4, regulatory subunit 2 | 0.35 | 6.85 | 0.20 | 6.85 | pre |
| 11722007_a_at | ENST00000589228.1 | NM_001276289, NM_001276290, NM_001278433, NM_002734, NM_212471, NM_212472, XM_011524983, XM_011524984, XM_011524985 | PRKAR1A | Protein kinase, camp-dependent, regulatory, type I, alpha | 0.34 | 9.93 | 0.23 | 9.93 | pre |
| 11753791_s_at | ENST00000589228.1 | NM_001276289, NM_001276290, NM_001278433, NM_002734, NM_212471, NM_212472, XM_011524983, XM_011524984, XM_011524985 | PRKAR1A | Protein kinase, camp-dependent, regulatory, type I, alpha | 0.39 | 8.04 | 0.25 | 8.04 | pre |
| 11723382_a_at | ENST00000376560.3 | NM_001077497, NM_025263, XM_011514922, XM_011547320, XM_011547721, XM_011547936, XM_011548107, XM_011548308, XM_011548491 | PRR3 | Proline rich 3 | 0.53 | 6.65 | 0.32 | 6.65 | pre |
| 11723383_a_at | ENST00000376560.3 | NM_001077497, NM_025263, XM_011514922, XM_011547320, XM_011547721, XM_011547936, XM_011548107, XM_011548308, XM_011548491 | PRR3 | Proline rich 3 | 0.48 | 6.81 | 0.45 | 6.81 | pre |
| 11762802_at | ENST00000412055.1 | NM_207351, XM_011533628, XM_011533629, XM_011533630, XR_245125 | PRRT3 | Proline-rich transmembrane protein 3 | 0.31 | 5.07 | 0.12 | 5.07 | pre |
| 11731897_a_at | ENST00000395872.1 | NM_002820, NM_198964, NM_198965, NM_198966, XM_011520774, XM_011520775 | PTHLH | Parathyroid hormone-like hormone | 0.61 | 5.28 | 0.64 | 5.28 | pre |
| 11717892_a_at | ENST00000370651.3 | NM_003463, XM_011536111, XM_011536112 | PTP4A1 | Protein tyrosine phosphatase type IVA, member 1 | 0.35 | 9.60 | 0.36 | 9.60 | pre |
| 11717897_a_at | ENST00000370651.3 | NM_003463, XM_011536111, XM_011536112 | PTP4A1 | Protein tyrosine phosphatase type IVA, member 1 | 0.36 | 10.10 | 0.42 | 10.10 | pre |
| 11746685_x_at | ENST00000463280.1 | NM_001289095, NM_001289096, NM_001289098, NM_001289099, NM_001289100, NM_001289101, NM_017771, XM_005265250, XM_005265252, XM_005265255, XM_005265256, XM_011533864, XM_011533865, XM_011533866, XM_011533867, XM_011533868, XR_940460, XR_940461, XR_940462, XR_940463 | PXK | PX domain containing serine/threonine kinase | 0.27 | 5.95 | 0.40 | 5.95 | pre |
| 11752622_a_at | ENST00000463280.1 | NM_001289095, NM_001289096, NM_001289098, NM_001289099, NM_001289100, NM_001289101, NM_017771, XM_005265250, XM_005265252, XM_005265255, XM_005265256, XM_011533864, XM_011533865, XM_011533866, XM_011533867, XM_011533868, XR_940460, XR_940461, XR_940462, XR_940463 | PXK | PX domain containing serine/threonine kinase | 0.38 | 6.12 | 0.31 | 6.12 | pre |
| 11752623_x_at | ENST00000463280.1 | NM_001289095, NM_001289096, NM_001289098, NM_001289099, NM_001289100, NM_001289101, NM_017771, XM_005265250, XM_005265252, XM_005265255, XM_005265256, XM_011533864, XM_011533865, XM_011533866, XM_011533867, XM_011533868, XR_940460, XR_940461, XR_940462, XR_940463 | PXK | PX domain containing serine/threonine kinase | 0.45 | 6.46 | 0.39 | 6.46 | pre |
| 11732559_at | ENST00000399300.2 | NM_203390, XM_005250915, XM_005250916, XM_011517027, XM_011517028, XM_011517029 | RBM12B | RNA binding motif protein 12B | 0.29 | 6.67 | 0.33 | 6.67 | pre |
| 11732560_s_at | ENST00000399300.2 | NM_203390, XM_005250915, XM_005250916, XM_011517027, XM_011517028, XM_011517029 | RBM12B | RNA binding motif protein 12B | 0.30 | 8.73 | 0.46 | 8.73 | pre |
| 11756273_a_at | ENST00000399300.2 | NM_203390, XM_005250915, XM_005250916, XM_011517027, XM_011517028, XM_011517029 | RBM12B | RNA binding motif protein 12B | 0.28 | 7.26 | 0.41 | 7.26 | pre |
| 11751756_a_at | NA | NM_001198836, NM_001198837, NM_006328, NM_032886 | RBM14 | RNA binding motif protein 14 | 0.26 | 8.72 | 0.42 | 8.72 | pre |
| 11743410_a_at | ENST00000261973.7 | NM_021239, XM_011537044, XM_011537045, XR_943501 | RBM25 | RNA binding motif protein 25 | 0.41 | 9.16 | 0.13 | 9.16 | pre |
| 11759711_a_at | ENST00000261973.7 | NM_021239, XM_011537044, XM_011537045, XR_943501 | RBM25 | RNA binding motif protein 25 | 0.37 | 6.66 | -0.21 | 6.66 | pre |
| 11763300_at | NA | NM_001198843, NM_001198844, NM_002896 | RBM4 | RNA binding motif protein 4 | 0.47 | 6.86 | -0.10 | 6.86 | pre |
| 11719571_a_at | ENST00000451788.1 | NM_001008925, NM_001009922, NM_001278536, NM_001278537, NM_001278538, NM_001278539, NM_015436, NR_037913, NR_037914, NR_103723, NR_103724, NR_103725, XM_011531838, XM_011531839 | RCHY1 | Ring finger and CHY zinc finger domain containing 1, E3 ubiquitin protein ligase | 0.34 | 8.32 | 0.35 | 8.32 | pre |
| 11722062_at | ENST00000373758.4 | NM_001001330, XM_011539501 | REEP3 | Receptor accessory protein 3 | 0.35 | 9.83 | 0.00 | 9.83 | pre |
| 11725079_a_at | ENST00000368323.3 | NM_001256820, NM_001256821, NM_006912 | RIT1 | Ras-like without CAAX 1 | 0.51 | 8.29 | 0.43 | 8.29 | pre |
| 11743344_a_at | ENST00000283632.4 | NM_022780 | RMND5A | Required for meiotic nuclear division 5 homolog A | 0.38 | 6.22 | 0.20 | 6.22 | pre |
| 11753427_a_at | ENST00000375734.2 | NM_001254738, NM_005168 | RND3 | Rho family gtpase 3 | 0.43 | 9.04 | 0.35 | 9.04 | pre |
| 11744116_s_at | ENST00000283646.4 | NM_144563, XR_939673 | RPIA | Ribose 5-phosphate isomerase A | 0.31 | 9.28 | 0.24 | 9.28 | pre |
| 11743270_a_at | ENST00000331738.7 | NM_023012, NM_198261, NM_198262, NR_036434, NR_036435, NR_036436, XM_005253601, XM_005253602, XM_005253604, XM_011538688, XM_011538689, XR_242958, XR_242959, XR_944681 | RSRC2 | Arginine/serine-rich coiled-coil 2 | 0.49 | 10.15 | 0.32 | 10.15 | pre |
| 11743271_s_at | ENST00000331738.7 | NM_023012, NM_198261, NM_198262, NR_036434, NR_036435, NR_036436, XM_005253601, XM_005253602, XM_005253604, XM_011538688, XM_011538689, XR_242958, XR_242959, XR_944681 | RSRC2 | Arginine/serine-rich coiled-coil 2 | 0.35 | 9.73 | 0.35 | 9.73 | pre |
| 11743272_a_at | ENST00000331738.7 | NM_023012, NM_198261, NM_198262, NR_036434, NR_036435, NR_036436, XM_005253601, XM_005253602, XM_005253604, XM_011538688, XM_011538689, XR_242958, XR_242959, XR_944681 | RSRC2 | Arginine/serine-rich coiled-coil 2 | 0.24 | 10.17 | 0.34 | 10.17 | pre |
| 11743274_x_at | ENST00000331738.7 | NM_023012, NM_198261, NM_198262, NR_036434, NR_036435, NR_036436, XM_005253601, XM_005253602, XM_005253604, XM_011538688, XM_011538689, XR_242958, XR_242959, XR_944681 | RSRC2 | Arginine/serine-rich coiled-coil 2 | 0.24 | 9.68 | 0.39 | 9.68 | pre |
| 11743490_a_at | ENST00000331738.7 | NM_023012, NM_198261, NM_198262, NR_036434, NR_036435, NR_036436, XM_005253601, XM_005253602, XM_005253604, XM_011538688, XM_011538689, XR_242958, XR_242959, XR_944681 | RSRC2 | Arginine/serine-rich coiled-coil 2 | 0.51 | 9.71 | 0.32 | 9.71 | pre |
| 11737535_at | ENST00000409003.4 | NM_001101676, NM_207506, NR_109794, XM_011517031, XM_011517032, XM_011517033, XM_011517034, XM_011517035, XM_011517036, XM_011517037, XR_928330, XR_928331, XR_928332, XR_928333 | SAMD12 | Sterile alpha motif domain containing 12 | 0.45 | 3.60 | 0.04 | 3.60 | pre |
| 11730102_at | ENST00000409672.1 | NM_002977, XM_005246757, XM_011511616, XM_011511617, XM_011511618, XM_011511619 | SCN9A | Sodium channel, voltage gated, type IX alpha subunit | 0.31 | 3.35 | 0.12 | 3.35 | pre |
| 11723721_a_at | ENST00000264313.6 | NM_020846, XM_005248121 | SLAIN2 | SLAIN motif family member 2 | 0.31 | 9.55 | 0.04 | 9.55 | pre |
| 11723723_at | ENST00000264313.6 | NM_020846, XM_005248121 | SLAIN2 | SLAIN motif family member 2 | 0.33 | 8.26 | 0.13 | 8.26 | pre |
| 11752963_a_at | ENST00000539591.1 | NM_001010875, NM_001286806, NM_001286807, XM_005266321, XM_005266322, XM_005266327, XM_006719793, XM_006719794, XM_011535026, XM_011535027, XM_011535028 | SLC25A30 | Solute carrier family 25, member 30 | 0.37 | 4.14 | -0.02 | 4.14 | pre |
| 11747964_a_at | ENST00000295736.5 | NM_001258379, NM_001258380, NM_003615, XM_005265598, XM_005265600, XM_005265601, XM_006713421, XM_011534255, XM_011534256, XM_011534257, XM_011534258, XM_011534259, XM_011534260, XM_011534261, XM_011534262, XM_011534263, XM_011534264, XM_011534265, XR_940517, XR_940518, XR_940519 | SLC4A7 | Solute carrier family 4, sodium bicarbonate cotransporter, member 7 | 0.43 | 8.46 | -0.09 | 8.46 | pre |
| 11744680_a_at | ENST00000280612.5 | NM_014331, XM_011531800, XM_011531801, XM_011531802, XM_011531803, XM_011531804, XM_011531805 | SLC7A11 | Solute carrier family 7 (anionic amino acid transporter light chain, xc- system), member 11 | 0.49 | 8.41 | 0.48 | 8.41 | pre |
| 11736318_a_at | ENST00000342415.5 | NM_152551, XM_011514312, XR_427825 | SNRNP48 | Small nuclear ribonucleoprotein, U11/U12 48kda subunit | 0.30 | 6.86 | 0.30 | 6.86 | pre |
| 11750248_x_at | ENST00000265909.4 | NM_001301089, NM_014758, XM_005271546, XM_011542819, XM_011542820, XM_011542821, XM_011542822, XM_011542823, XM_011542824, XM_011542825, XM_011542826, XM_011542827, XR_428976, XR_428977 | SNX19 | Sorting nexin 19 | 0.36 | 5.23 | 0.17 | 5.23 | pre |
| 11726179_a_at | ENST00000316399.6 | NM_001145811, NM_001145819, NM_017508, NM_033326 | SOX6 | SRY box 6 | 0.35 | 3.44 | 0.15 | 3.44 | pre |
| 11727811_a_at | ENST00000258962.4 | NM_001078166, NM_006924, NR_034041, XM_006722012, XR_429911, XR_429912 | SRSF1 | Serine/arginine-rich splicing factor 1 | 0.44 | 10.73 | 0.24 | 10.73 | pre |
| 11747684_a_at | ENST00000258962.4 | NM_001078166, NM_006924, NR_034041, XM_006722012, XR_429911, XR_429912 | SRSF1 | Serine/arginine-rich splicing factor 1 | 0.35 | 10.88 | 0.23 | 10.88 | pre |
| 11744850_a_at | NA | NM_001282129, NM_001282130, NM_001282131, NM_033389, XM_005258058, XM_005258059, XM_005258060, XM_006722149, XM_011525402, XM_011525403, XM_011525404, XM_011525405, XM_011525406, XM_011525407 | SSH2 | Slingshot protein phosphatase 2 | 0.43 | 6.13 | 0.53 | 6.13 | pre |
| 11763451_s_at | ENST00000291386.3 | NM_014188 | SSU72 | SSU72 homolog, RNA polymerase II CTD phosphatase | 0.31 | 7.45 | 0.40 | 7.45 | pre |
| 11720598_x_at | ENST00000265073.4 | NM_006713, XM_011513943, XM_011513944 | SUB1 | SUB1 homolog, transcriptional regulator | 0.31 | 10.33 | 0.12 | 10.33 | pre |
| 11717623_a_at | ENST00000367723.4 | NM_001282750, NM_001282751, NM_014283, NM_016227, XM_006711374, XM_006711375, XM_006711376, XR_921826 | SUCO | SUN domain containing ossification factor | 0.27 | 7.64 | 0.42 | 7.64 | pre |
| 11730540_a_at | ENST00000543857.1 | NM_001201536, NM_005681, NM_139352, XM_005273343, XM_006711612, XM_006711613, XM_011510112 | TAF1A | TATA box binding protein (TBP)-associated factor, RNA polymerase I, A, 48kda | 0.35 | 6.86 | 0.40 | 6.86 | pre |
| 11730541_x_at | ENST00000543857.1 | NM_001201536, NM_005681, NM_139352, XM_005273343, XM_006711612, XM_006711613, XM_011510112 | TAF1A | TATA box binding protein (TBP)-associated factor, RNA polymerase I, A, 48kda | 0.32 | 7.90 | 0.42 | 7.90 | pre |
| 11747455_a_at | ENST00000543857.1 | NM_001201536, NM_005681, NM_139352, XM_005273343, XM_006711612, XM_006711613, XM_011510112 | TAF1A | TATA box binding protein (TBP)-associated factor, RNA polymerase I, A, 48kda | 0.47 | 6.14 | 0.45 | 6.14 | pre |
| 11760914_x_at | ENST00000543857.1 | NM_001201536, NM_005681, NM_139352, XM_005273343, XM_006711612, XM_006711613, XM_011510112 | TAF1A | TATA box binding protein (TBP)-associated factor, RNA polymerase I, A, 48kda | 0.32 | 6.14 | 0.26 | 6.14 | pre |
| 11718053_a_at | ENST00000344949.5 | NM_001199198, NM_018309, XM_011512974 | TBC1D23 | TBC1 domain family, member 23 | 0.36 | 8.57 | 0.07 | 8.57 | pre |
| 11748475_a_at | ENST00000296702.5 | NM_001040006, NM_006706, XM_005268365, XM_011537545, XM_011537546, XM_011537547, XM_011537548, XM_011537549, XR_944306, XR_944307 | TCERG1 | Transcription elongation regulator 1 | 0.38 | 8.72 | 0.11 | 8.72 | pre |
| 11750840_s_at | ENST00000359013.4 | NM_001024847, NM_003242, XM_011534043, XM_011534044, XM_011534045 | TGFBR2 | Transforming growth factor beta receptor II | 0.41 | 7.85 | 0.08 | 7.85 | pre |
| 11733906_a_at | ENST00000345117.2 | NM_018105, NM_199003 | THAP1 | THAP domain containing, apoptosis associated protein 1 | 0.40 | 8.46 | 0.36 | 8.46 | pre |
| 11719934_a_at | ENST00000462138.1 | NM_001064, NM_001135055, NM_001135056, NM_001258028, NR_047579, NR_047580, XM_011534054, XM_011534055 | TKT | Transketolase | 0.68 | 7.07 | 0.63 | 7.07 | pre |
| 11761536_a_at | NA | NM_001307960, NM_001308026, NM_025141, NM_078474, XM_005254980, XM_005254981 | TM2D3 | TM2 domain containing 3 | 0.48 | 3.05 | 0.27 | 3.05 | pre |
| 11751628_a_at | ENST00000275767.3 | NM_018295 | TMEM140 | Transmembrane protein 140 | 0.34 | 5.11 | 0.05 | 5.11 | pre |
| 11716229_s_at | ENST00000338272.8 | NM_020141, XM_011541796 | TMEM167B | Transmembrane protein 167B | 0.33 | 7.39 | 0.26 | 7.39 | pre |
| 11758747_at | ENST00000338272.8 | NM_020141, XM_011541796 | TMEM167B | Transmembrane protein 167B | 0.32 | 3.43 | 0.10 | 3.43 | pre |
| 11758287_s_at | ENST00000377495.1 | NM_001013629, NM_001098844, XM_011519623, XM_011519624, XM_011519625, XM_011519626, XM_011519627, XM_011519628, XM_011519629 | TMEM236 | Transmembrane protein 236 | 0.48 | 5.74 | 0.18 | 5.74 | pre |
| 11758287_s_at | ENST00000480516.1 | NM_001013629, NM_001098844, XM_011519623, XM_011519624, XM_011519625, XM_011519626, XM_011519627, XM_011519628, XM_011519629 | TMEM236 | Transmembrane protein 236 | 0.48 | 5.74 | 0.18 | 5.74 | pre |
| 11719419_a_at | ENST00000415050.2 | NM_001098621, NM_015676 | TMEM251 | Transmembrane protein 251 | 0.36 | 9.03 | 0.26 | 9.03 | pre |
| 11716891_at | ENST00000358227.4 | NM_033428, XM_011518062 | TMEM261 | Transmembrane protein 261 | 0.31 | 5.72 | 0.05 | 5.72 | pre |
| 11758682_s_at | ENST00000358227.4 | NM_033428, XM_011518062 | TMEM261 | Transmembrane protein 261 | 0.36 | 5.60 | 0.39 | 5.60 | pre |
| 11756750_a_at | ENST00000555868.1 | NM_001017970 | TMEM30B | Transmembrane protein 30B | 0.31 | 7.60 | 0.23 | 7.60 | pre |
| 11756193_a_at | ENST00000302392.4 | NM_144638, XR_940376, XR_953189 | TMEM42 | Transmembrane protein 42 | 0.27 | 8.62 | 0.39 | 8.62 | pre |
| 11763744_at | ENST00000453321.3 | NM_001142301, NM_153704, NR_024522, XM_006716686, XM_006716687, XM_011517363, XR_428387, XR_928360, XR_928361, XR_928362 | TMEM67 | Transmembrane protein 67 | 0.40 | 3.65 | 0.25 | 3.65 | pre |
| 11760192_s_at | ENST00000523073.1 | NM_001286657, NM_001286660, NM_001286661, NM_152417, XM_005251150, XM_011517454, XM_011517455 | TMEM68 | Transmembrane protein 68 | 0.29 | 8.38 | 0.45 | 8.38 | pre |
| 11727484_a_at | ENST00000367478.4 | NM_003292, XM_011509955 | TPR | Translocated promoter region, nuclear basket protein | 0.33 | 8.17 | -0.05 | 8.17 | pre |
| 11724261_a_at | ENST00000344204.4 | NM_007118, XM_011514107, XM_011514108, XM_011514109, XM_011514110, XM_011514111, XM_011514112, XM_011514113, XR_241714 | TRIO | Trio Rho guanine nucleotide exchange factor | 1.30 | 7.46 | 0.74 | 7.46 | pre |
| 11744590_a_at | ENST00000344204.4 | NM_007118, XM_011514107, XM_011514108, XM_011514109, XM_011514110, XM_011514111, XM_011514112, XM_011514113, XR_241714 | TRIO | Trio Rho guanine nucleotide exchange factor | 0.40 | 8.14 | 0.14 | 8.14 | pre |
| 11745452_a_at | ENST00000370141.2 | NM_019083, XM_005270945, XM_005270946, XR_246273, XR_246274, XR_246275, XR_246276 | TRMT13 | Trna methyltransferase 13 homolog (S. Cerevisiae) | 0.53 | 5.44 | 0.28 | 5.44 | pre |
| 11745453_x_at | ENST00000370141.2 | NM_019083, XM_005270945, XM_005270946, XR_246273, XR_246274, XR_246275, XR_246276 | TRMT13 | Trna methyltransferase 13 homolog (S. Cerevisiae) | 0.50 | 6.96 | 0.32 | 6.96 | pre |
| 11746421_a_at | ENST00000370141.2 | NM_019083, XM_005270945, XM_005270946, XR_246273, XR_246274, XR_246275, XR_246276 | TRMT13 | Trna methyltransferase 13 homolog (S. Cerevisiae) | 0.55 | 7.75 | 0.26 | 7.75 | pre |
| 11760405_x_at | ENST00000370141.2 | NM_019083, XM_005270945, XM_005270946, XR_246273, XR_246274, XR_246275, XR_246276 | TRMT13 | Trna methyltransferase 13 homolog (S. Cerevisiae) | 0.43 | 7.64 | 0.25 | 7.64 | pre |
| 11761560_x_at | ENST00000370141.2 | NM_019083, XM_005270945, XM_005270946, XR_246273, XR_246274, XR_246275, XR_246276 | TRMT13 | Trna methyltransferase 13 homolog (S. Cerevisiae) | 0.40 | 8.00 | 0.22 | 8.00 | pre |
| 11748214_a_at | ENST00000361875.3 | NM_001303264, NM_014779, NR_130136, XM_011513337, XM_011513338, XM_011513339, XR_427388, XR_924223 | TSC22D2 | TSC22 domain family, member 2 | 0.42 | 8.15 | 0.18 | 8.15 | pre |
| 11750085_a_at | ENST00000361875.3 | NM_001303264, NM_014779, NR_130136, XM_011513337, XM_011513338, XM_011513339, XR_427388, XR_924223 | TSC22D2 | TSC22 domain family, member 2 | 0.36 | 7.32 | 0.24 | 7.32 | pre |
| 11744760_s_at | ENST00000379706.4 | NM_033035, NM_138551, NR_045089, XM_011543698 | TSLP | Thymic stromal lymphopoietin | 0.47 | 3.41 | 0.53 | 3.41 | pre |
| 11715650_a_at | ENST00000378070.4 | NM_001093771, NM_001261445, NM_001261446, NM_003330, NM_182729, NM_182742, NM_182743 | TXNRD1 | Thioredoxin reductase 1 | 0.37 | 11.29 | 0.33 | 11.29 | pre |
| 11750416_a_at | ENST00000378070.4 | NM_001093771, NM_001261445, NM_001261446, NM_003330, NM_182729, NM_182742, NM_182743 | TXNRD1 | Thioredoxin reductase 1 | 0.34 | 11.15 | 0.34 | 11.15 | pre |
| 11750058_a_at | ENST00000517608.1 | NM_001001481, NM_001001482, NM_001271015, NM_018299, NR_073119, NR_073120, NR_073121 | UBE2W | Ubiquitin-conjugating enzyme E2W (putative) | 0.47 | 7.27 | 0.20 | 7.27 | pre |
| 11744582_a_at | ENST00000353364.3 | NM_001252078, NM_001252079, NM_006313, XM_005269259, XM_005269261, XM_006719718, XR_944851 | USP15 | Ubiquitin specific peptidase 15 | 0.30 | 4.93 | 0.15 | 4.93 | pre |
| 11746454_a_at | ENST00000353364.3 | NM_001252078, NM_001252079, NM_006313, XM_005269259, XM_005269261, XM_006719718, XR_944851 | USP15 | Ubiquitin specific peptidase 15 | 0.33 | 8.12 | 0.15 | 8.12 | pre |
| 11762389_at | ENST00000307017.4 | NM_001290325, NM_001290326, NM_032557, XM_011532360 | USP38 | Ubiquitin specific peptidase 38 | 0.70 | 4.26 | 0.66 | 4.26 | pre |
| 11731502_at | ENST00000309822.2 | NM_032334, XM_005251080, XR_928356, XR_928357 | UTP23 | UTP23, small subunit (SSU) processome component, homolog (yeast) | 0.41 | 8.14 | 0.19 | 8.14 | pre |
| 11757631_s_at | ENST00000398399.2 | NM_016206, XM_005264752, XM_006713138, XM_006713139 | VGLL3 | Vestigial-like family member 3 | 0.34 | 7.29 | 0.06 | 7.29 | pre |
| 11717284_a_at | ENST00000223023.4 | NM_003941 | WASL | Wiskott-Aldrich syndrome-like | 0.41 | 8.54 | 0.00 | 8.54 | pre |
| 11717285_a_at | ENST00000223023.4 | NM_003941 | WASL | Wiskott-Aldrich syndrome-like | 0.31 | 5.89 | 0.28 | 5.89 | pre |
| 11751592_a_at | ENST00000349139.5 | NM_006784 | WDR3 | WD repeat domain 3 | 0.55 | 6.65 | 0.18 | 6.65 | pre |
| 11723738_a_at | ENST00000506538.2 | NM_139281, XM_011543163 | WDR36 | WD repeat domain 36 | 0.34 | 8.01 | 0.10 | 8.01 | pre |
| 11762563_at | ENST00000382895.3 | NM_001042424, NM_007331, NM_014919, NM_133330, NM_133331, NM_133332, NM_133333, NM_133334, NM_133335, NM_133336, XM_005248001, XM_005248002, XM_005248005, XM_006713914, XM_006713915, XM_011513557, XM_011513558, XM_011513559, XM_011513560 | WHSC1 | Wolf-Hirschhorn syndrome candidate 1 | 0.31 | 4.72 | 0.12 | 4.72 | pre |
| 11737175_at | ENST00000315939.6 | NM_001184985, NM_014823, NM_018979, NM_213655, XM_006719003, XM_011520997, XM_011520998, XM_011520999, XM_011521000, XM_011521001, XM_011521002, XM_011521003, XM_011521004, XM_011521005, XM_011521006, XM_011521007, XM_011521008, XM_011521009 | WNK1 | WNK lysine deficient protein kinase 1 | 0.44 | 4.48 | 0.06 | 4.48 | pre |
| 11743280_a_at | ENST00000315939.6 | NM_001184985, NM_014823, NM_018979, NM_213655, XM_006719003, XM_011520997, XM_011520998, XM_011520999, XM_011521000, XM_011521001, XM_011521002, XM_011521003, XM_011521004, XM_011521005, XM_011521006, XM_011521007, XM_011521008, XM_011521009 | WNK1 | WNK lysine deficient protein kinase 1 | 0.33 | 8.86 | -0.03 | 8.86 | pre |
| 11762793_at | ENST00000264335.8 | NM_006761, NR_024058, XM_005256784, XM_006725298 | YWHAE | Tyrosine 3-monooxygenase/tryptophan 5-monooxygenase activation protein, epsilon | 0.31 | 4.87 | 0.27 | 4.87 | pre |
| 11749652_a_at | ENST00000398505.3 | NM_001098402, NM_001098403, NM_020727, XM_005261121, XM_005261123, XM_011529587, XM_011529588, XM_011529589, XM_011529590, XM_011529591, XM_011529592 | ZBTB21 | Zinc finger and BTB domain containing 21 | 0.26 | 5.00 | 0.39 | 5.00 | pre |
| 11758414_s_at | ENST00000398505.3 | NM_001098402, NM_001098403, NM_020727, XM_005261121, XM_005261123, XM_011529587, XM_011529588, XM_011529589, XM_011529590, XM_011529591, XM_011529592 | ZBTB21 | Zinc finger and BTB domain containing 21 | 0.30 | 7.35 | 0.45 | 7.35 | pre |
| 11729725_at | ENST00000373659.3 | NM_006626 | ZBTB6 | Zinc finger and BTB domain containing 6 | 0.37 | 5.50 | 0.27 | 5.50 | pre |
| 11736118_a_at | ENST00000237937.3 | NM_001102420, NM_001102421, NM_001278243, NM_001278244, NM_001278245, NM_006007 | ZFAND5 | Zinc finger, AN1-type domain 5 | 0.43 | 7.57 | 0.28 | 7.57 | pre |
| 11743753_x_at | ENST00000237937.3 | NM_001102420, NM_001102421, NM_001278243, NM_001278244, NM_001278245, NM_006007 | ZFAND5 | Zinc finger, AN1-type domain 5 | 0.41 | 7.49 | 0.21 | 7.49 | pre |
| 11743755_s_at | ENST00000237937.3 | NM_001102420, NM_001102421, NM_001278243, NM_001278244, NM_001278245, NM_006007 | ZFAND5 | Zinc finger, AN1-type domain 5 | 0.39 | 7.93 | 0.24 | 7.93 | pre |
| 11762631_a_at | ENST00000378743.3 | NM_144982 | ZFC3H1 | Zinc finger, C3H1-type containing | 0.78 | 4.48 | 0.94 | 4.48 | pre |
| 11762632_at | ENST00000378743.3 | NM_144982 | ZFC3H1 | Zinc finger, C3H1-type containing | 0.78 | 4.12 | 0.51 | 4.12 | pre |
| 11737829_at | ENST00000330236.6 | NM_001278119, NM_001278121, NM_001278122, NM_006298, XM_011514870, XM_011514871, XM_011514872, XM_011514873, XM_011514874 | ZKSCAN8 | Zinc finger with KRAB and SCAN domains 8 | 0.39 | 3.56 | 0.07 | 3.56 | pre |
| 11739968_a_at | NA | NM_001300949, NM_033204, XM_006722942 | ZNF101 | Zinc finger protein 101 | 0.50 | 5.78 | 0.48 | 5.78 | pre |
| 11751139_x_at | NA | NM_001300949, NM_033204, XM_006722942 | ZNF101 | Zinc finger protein 101 | 0.37 | 5.63 | 0.31 | 5.63 | pre |
| 11731497_a_at | ENST00000396161.5 | NM_003435, XM_011508876, XM_011508877 | ZNF134 | Zinc finger protein 134 | 0.32 | 7.01 | 0.30 | 7.01 | pre |
| 11731498_a_at | ENST00000396161.5 | NM_003435, XM_011508876, XM_011508877 | ZNF134 | Zinc finger protein 134 | 0.30 | 6.70 | 0.36 | 6.70 | pre |
| 11759239_at | ENST00000240499.7 | NM_003441, XM_011513562, XM_011513563 | ZNF141 | Zinc finger protein 141 | 0.26 | 5.04 | 0.31 | 5.04 | pre |
| 11743472_a_at | ENST00000588883.1 | NM_001032372, NM_001032373, NM_001032374, NM_001032375, NM_001146220, NM_015919, NM_016444, XM_005259224, XM_005259225, XM_005259226, XM_005259227, XM_005259228, XM_006723367, XM_006723368, XM_006723369, XM_011527288 | ZNF226 | Zinc finger protein 226 | 0.39 | 7.48 | 0.29 | 7.48 | pre |
| 11743473_x_at | ENST00000588883.1 | NM_001032372, NM_001032373, NM_001032374, NM_001032375, NM_001146220, NM_015919, NM_016444, XM_005259224, XM_005259225, XM_005259226, XM_005259227, XM_005259228, XM_006723367, XM_006723368, XM_006723369, XM_011527288 | ZNF226 | Zinc finger protein 226 | 0.32 | 7.55 | 0.40 | 7.55 | pre |
| 11732421_at | ENST00000426739.2 | NM_001144824, NM_006630, XM_006722974, XM_011526323, XM_011526324 | ZNF234 | Zinc finger protein 234 | 0.33 | 6.64 | -0.17 | 6.64 | pre |
| 11739672_x_at | ENST00000589717.1 | NM_021047, XM_011528134, XM_011528135 | ZNF253 | Zinc finger protein 253 | 0.40 | 5.37 | 0.21 | 5.37 | pre |
| 11749641_x_at | ENST00000357002.4 | NM_001278661, NM_001278662, NM_001278663, NM_001278664, NM_001278665, NM_001278677, NM_001278678, NM_004876, NM_203282, XM_006722949, XM_011528443, XM_011528444, XM_011528445, XM_011528446, XM_011528447, XM_011528448 | ZNF254 | Zinc finger protein 254 | 0.53 | 5.87 | 0.49 | 5.87 | pre |
| 11717868_a_at | NA | NM_005741, XM_005255031, XM_006720831, XM_006720832, XM_006720833, XM_006720834, XM_006720835, XM_011522344 | ZNF263 | Zinc finger protein 263 | 0.54 | 7.23 | 0.29 | 7.23 | pre |
| 11717869_at | NA | NM_005741, XM_005255031, XM_006720831, XM_006720832, XM_006720833, XM_006720834, XM_006720835, XM_011522344 | ZNF263 | Zinc finger protein 263 | 0.52 | 8.52 | 0.31 | 8.52 | pre |
| 11717870_x_at | NA | NM_005741, XM_005255031, XM_006720831, XM_006720832, XM_006720833, XM_006720834, XM_006720835, XM_011522344 | ZNF263 | Zinc finger protein 263 | 0.51 | 8.23 | 0.26 | 8.23 | pre |
| 11751038_a_at | NA | NM_005741, XM_005255031, XM_006720831, XM_006720832, XM_006720833, XM_006720834, XM_006720835, XM_011522344 | ZNF263 | Zinc finger protein 263 | 0.54 | 7.11 | 0.28 | 7.11 | pre |
| 11727522_a_at | ENST00000300870.10 | NM_001265588, NM_003414, NR_049749 | ZNF267 | Zinc finger protein 267 | 0.34 | 7.45 | 0.49 | 7.45 | pre |
| 11727523_x_at | ENST00000300870.10 | NM_001265588, NM_003414, NR_049749 | ZNF267 | Zinc finger protein 267 | 0.40 | 8.39 | 0.51 | 8.39 | pre |
| 11754869_s_at | ENST00000300870.10 | NM_001265588, NM_003414, NR_049749 | ZNF267 | Zinc finger protein 267 | 0.44 | 7.17 | 0.49 | 7.17 | pre |
| 11760921_a_at | ENST00000370708.4 | NM_001031623, NM_001257273, NM_015555, XM_005248994, XM_011514460, XM_011514461, XM_011514462, XM_011514463, XR_241885 | ZNF451 | Zinc finger protein 451 | 0.43 | 5.65 | 0.16 | 5.65 | pre |
| 11740309_a_at | ENST00000360338.3 | NM_006635, XM_005258422 | ZNF460 | Zinc finger protein 460 | 0.42 | 3.50 | 0.28 | 3.50 | pre |
| 11740310_a_at | ENST00000360338.3 | NM_006635, XM_005258422 | ZNF460 | Zinc finger protein 460 | 0.32 | 3.85 | 0.37 | 3.85 | pre |
| 11732294_a_at | ENST00000454319.1 | NM_001145343, NM_001145344, NM_001145345, NM_001300970, NM_032838, XM_005259354, XM_005259356, XM_006723447, XM_011527428, XM_011527429, XM_011527430 | ZNF566 | Zinc finger protein 566 | 0.51 | 5.82 | 0.29 | 5.82 | pre |
| 11755505_a_at | ENST00000486655.1 | NM_001042697, NM_001042698 | ZSWIM7 | Zinc finger, SWIM-type containing 7 | 0.44 | 7.65 | 0.28 | 7.65 | pre |
