# Supplementary material for: Silencing of miR-182 is associated with modulation of tumorigenesis through apoptosis induction in an experimental model of colorectal cancer
Source: BMC Cancer. 2019 Aug 20;19:821. doi: 10.1186/s12885-019-5982-9 (PMC6700772; doi:10.1186/s12885-019-5982-9)
Supplement: Supplementary file 3 — Table S3. Gene Ontology (GO) functional pathways significantly enriched considering 1382 genes differentially expressed after miR-182 inhibition in MICOL-14tum cells. BP, Biological Process; CC, Cellular Component; MF, Molecular Function. (DOCX 25 kb) [file 12885_2019_5982_MOESM3_ESM.docx]

**Supplementary Table 3. Gene Ontology (GO) functional pathways significantly enriched considering 1382 genes differentially expressed after miR-182 inhibition in MICOL-14^tum^ cells.** BP, Biological Process; CC, Cellular Component; MF, Molecular Function.

| **Functional category** | **Term/Pathway** | **Gene symbol** | **Genes** | **Fold Enrichment** | **Adjusted p-value** |
| --- | --- | --- | --- | --- | --- |
| GO BP | GO:0000278~mitotic cell cycle | ITGB3BP, CEP72, E2F3, CEP78, NUP188, FER, HIST2H4A, MCM10, CCNE2, CCNE1, BLZF1, RAE1, CDKN2C, ORC5, MASTL, TPR, TOP2A, CCNA2, ORC3, CDK1, DSN1, ESPL1, OPTN, NUPL2, ESCO2, CDK2, RFC5, FGFR1OP, NSL1, RRM2, USO1, BUB1B, NUP43, ANAPC16, USP3, POLA1, AZI2, NDC1, TYMS, TUBGCP3, TUBGCP5, POLE2, NCAPG, HIST1H4E, FBXO5, FBXW11, FEN1, ERCC6L, CENPO, RAB2A, GINS1, CENPN, PDS5B, KIF18A, CDC23, CASC5, NDC80, CENPE, BIRC5, SMC2, CENPI, CDC25A, SMC3, SMC4, PLK4, CDKN1A, PSMC4, PSMD12, CENPU, SMC1A | 70 | 2.33 | 2.03E-07 |
| GO BP | GO:0006281~DNA repair | COPS2, CLSPN, RAD51C, NBN, HIST2H4A, BOD1L1, FANCI, H2AFX, CDK1, DTL, UFD1L, USP1, LIG3, TOPBP1, MBD4, POLB, RAD52, RMI1, CDK2, RFC5, UBE2N, NABP1, XPC, FANCD2, UBE2W, GADD45A, PPP4R2, EID3, BLM, USP3, POLR2K, SLF2, SLF1, KIAA0101, POLA1, CHEK1, FAAP20, SUMO3, ERCC8, POLE2, HIST1H4E, TCEA1, ACTL6A, RCHY1, FEN1, ERCC2, EXO1, RAD51AP1, MSH2, BRIP1, WHSC1, RAD54L, BRCA1, SMC3, ATRX, PARPBP, BRE, ZRANB3, CUL4B, SMC1A, ALKBH3 | 61 | 2.28 | 2.82E-06 |
| GO BP | GO:0051301~cell division | ITGB3BP, SENP5, LATS1, CCNE2, CCNE1, CCSAP, NSMCE2, MASTL, TPR, CCNA2, KIF14, CDK1, KIF11, DSN1, LIG3, HMGA2, CDK2, TACC1, CHMP1B, SYCP3, NSL1, MCMBP, BUB1B, MAPRE2, ARL8B, NUP43, SEPT9, ANAPC16, HAUS6, MPLKIP, USP9X, NCAPG, FBXO5, TNKS, NSUN2, HELLS, ERCC6L, CSNK1A1, PARD6B, PDS5B, CDC23, CASC5, NDC80, BIRC5, CENPE, SMC2, CDC25A, SMC3, SMC4, ANXA11, CENPV, KIF20B, BRE, MIS18BP1, SMC1A | 55 | 2.41 | 4.23E-06 |
| GO BP | GO:0007067~mitotic nuclear division | ITGB3BP, ANAPC16, HAUS6, MPLKIP, USP9X, LATS1, CCSAP, NSMCE2, TNKS, MASTL, TPR, NSUN2, CCNA2, HELLS, ASPM, ERCC6L, CSNK1A1, CDK1, CENPN, KIF11, DSN1, KIF15, CDC23, CASC5, NDC80, BIRC5, GEM, HMGA2, CDK2, CDC25A, SMC3, SYCP3, NSL1, MCMBP, BRE, KIF20B, CENPV, BUB1B, MAPRE2, MIS18BP1, NUP43 | 41 | 2.41 | 2.30E-04 |
| GO BP | GO:0000724~double-strand break repair via homologous recombination | CLSPN, RAD51C, NBN, PPP4R2, BLM, CHEK1, HIST2H4A, SFR1, HIST1H4E, NSMCE2, H2AFX, FEN1, EXO1, RAD51AP1, YY1, LIG3, ZSWIM7, BRIP1, WHSC1, TOPBP1, RAD52, RAD54L, RMI1, BRCA1, CDK2, UBE2N, RFC5, NABP1, SFPQ, BRE | 30 | 3.03 | 1.12E-04 |
| GO BP | GO:0006302~double-strand break repair | CLSPN, RAD51C, NBN, PPP4R2, BLM, KIAA0430, CHEK1, HIST2H4A, HIST1H4E, H2AFX, FEN1, TRIP13, EXO1, RAD51AP1, MSH2, LIG3, BRIP1, WHSC1, TOPBP1, RAD52, RMI1, ESCO2, BRCA1, CDK2, UBE2N, RFC5, BRE | 27 | 2.54 | 7.58E-03 |
| GO BP | GO:0006260~DNA replication | CLSPN, KIAA0101, NAP1L1, POLA1, CHEK1, MCM10, TOP1, POLE2, ORC5, FEN1, RBMS1, ORC3, CDK1, DTL, RMI1, RBBP6, CDK2, BRCA1, CDC25A, RFC5, RRM2, TBRG1, NFIC, NFIA, DUT | 25 | 2.71 | 6.20E-03 |
| GO BP | GO:0016925~protein sumoylation | EID3, BLM, ZNF451, SAE1, BIRC5, NUP188, RAD52, SENP5, NUPL2, SMC3, BRCA1, NFATC2IP, NDC1, SUMO3, PHC3, TOP1, XPC, RAE1, NSMCE2, TPR, SMC1A, TOP2A, NUP43 | 23 | 2.87 | 7.01E-03 |
| GO BP | GO:0007059~chromosome segregation | CENPN, KIF11, DSN1, USP9X, NDC80, BIRC5, CENPE, BRCA1, ESCO2, CIAO1, TOP1, NSL1, ARL8B, BRD4, TOP2A, NUP43, ERCC2 | 17 | 3.31 | 0.0139 |
| GO BP | GO:0007095~mitotic G2 DNA damage checkpoint | CDK1, NBN, BLM, FANCI, SYF2, HMGA2, CCNA2 | 7 | 7.66 | 0.0444 |
| GO CC | GO:0005737~cytoplasm | TUBB2A, NAA15, NAA16, STRN, PNISR, CLK1, ZNF638, SART3, CDCA7, HIST1H2BN, RAE1, CCNA2, PLS3, KRR1, IKBKAP, MAGI2, ZHX2, ESPL1, MECOM, RPTOR, ERGIC2, DCAF7, PTRF, MAPK6, KIAA1524, ARL8B, CRTC2, ACP6, NTAN1, UBA5, BCL2L1, ACP1, NAA35, PDSS2, ADAP1, SH3BP5L, RILPL1, RAC1, RCHY1, C19ORF24, TCF3, RANBP17, ZC3H15, MKI67, ACACA, GARS, FNIP1, FNIP2, UPF3A, SRSF3, SRSF5, AIDA, WDR61, PARPBP, PKP4, CDC42BPA, TGFBR3, PPP2R3C, SRSF1, CREBRF, HSF2, EEF2K, ANP32A, EPG5, MASTL, BRD4, TARSL2, USP15, ATF7IP, RAP2C, ERLIN2, KIAA0586, CCT6A, RFTN1, FMN1, PJA2, FANCD2, RRM2, OSBPL10, TXK, ZNF480, USP24, ARL4A, SRGAP2, SNAP29, EXOC8, POLA1, EPB41L4A, ERI1, ZNF655, MTMR2, SRRT, LRRTM4, DGKE, MTMR9, PER3, SNAP23, ACSL4, ZNF263, HIST1H2BD, PPHLN1, NUCKS1, HIST1H2BG, ATRN, CAPN2, APPL2, RPL28, MID2, RPS6KA3, DUSP3, RPL22, GSK3B, SFPQ, DYM, USP48, MPHOSPH6, KATNAL1, MPHOSPH8, SRP14, METTL21B, AP1G1, EIF5, RBM4, STYX, IL11, ATAT1, CDKN2C, DNAJC9, BPNT1, ASPM, EGFR, BCL10, RBFOX2, TWF1, RELB, RPS6KC1, TOPBP1, FADD, PKIA, TACC1, NABP1, HIF1A, HSPB8, STMN1, ACTBL2, EID1, ARFGAP3, MPLKIP, EID3, STAM2, SLF1, NHS, SESN2, AZI2, AKT1S1, PTK6, PRKRA, GMPPA, FBXW2, RPL4, ARHGDIB, ERCC6L, GINS1, PHACTR4, BRIP1, WHSC1, WIPI1, SAFB2, PRKAR1A, LVRN, ZNF318, FBXO32, HSPD1, CYB5R3, E2F3, GPBP1, ALG2, ANO1, DICER1, RPRM, FER, NBPF1, CASP8, LRWD1, CNTLN, KDM5B, CASP2, NFX1, CDK1, ARL1, DENND6A, KIF11, NUSAP1, IRF2BP2, ZFR, CHAMP1, CDK2, SDC1, TRNAU1AP, FAM120A, DDT, MAPRE2, EMC2, SNX19, HYPK, SLC7A6OS, HAUS6, HYLS1, GDAP1, RPS15A, NR3C1, NDC1, TSC22D1, CHD9, NSUN2, PPP4R3B, EXO1, WDFY3, BBOF1, NF1, RCAN1, RCAN3, ANXA5, UBE2Q2, SMC2, ANXA3, SMC3, SMC4, INVS, ZBED4, AKR1B1, JAK2, CUL4B, ALKBH3, ITGB3BP, RAB3GAP2, RAD51C, TMEM18, DZIP3, ALOXE3, PDLIM7, PTPN21, AQP7, HOOK3, G2E3, CD44, WWP2, RAB23, EIF2B2, YY1, WNK1, KRT10, POLB, OPTN, FARP1, TANK, CEP350, CDCA7L, EPS8L2, COASY, SRSF10, IGF2BP2, AFAP1L1, PXK, TIPRL, VRK2, AGGF1, HOXA10, FBXO5, KLF5, MSTO1, SMYD3, NDFIP2, SMYD2, NOTCH3, FAM101B, RNF7, C1ORF116, CCT8, GRK5, CLSPN, COPS2, WASF3, CPEB2, FERMT2, VPS37A, SHOC2, CAMKK2, PARN, BLZF1, FANCI, ANKRD11, AGAP1, FAM129A, CEP112, RBM44, DSN1, HERC6, MBD1, RBBP6, DNAJC24, FCRLB, GADD45B, GADD45A, PLEK2, USP3, CEP126, PPFIA1, FKBP1A, CEP128, MLF1, PLCL2, SUMO3, GALK2, STK40, SQSTM1, NCAPG, DTNB, KIF21A, SPATA5, MLLT4, RBM25, PLEC, UBXN1, BIRC5, HNRNPDL, RAPH1, COG3, PAPOLA, C4ORF46, SLC16A7, PSMD12, PYGL, KIF20B, APBB2, ATP6V0A2, CALM1, STIL, ZC3HAV1, EIF5B, PI4K2B, MCM10, SMNDC1, GSTM3, MCOLN3, ZNF185, ORC5, MLKL, PDRG1, CUTC, NQO2, CEP89, DTL, RRP8, NUPL2, CDKL3, STK4, ELL2, PTHLH, TNS3, MCMBP, SLU7, RIPK4, CSNK1G3, DST, MAP7D3, SEPT9, ANAPC16, ZFAND5, BLM, PNPT1, SOX4, SNX4, CMPK1, RASAL2, BLOC1S4, CSE1L, MFAP3L, STRBP, RNF14, CFLAR, PARD6B, GABARAPL2, RPGRIP1L, CASC5, BRCA1, RIMKLB, UACA, PSMC4, SLAIN2, ITGA7, WDR4, CPNE3, TMPO, FPGS, SMC1A, PDCD6, C9ORF72, HECW2, CEP57L1, PDCD2, NFATC2IP, SLK, MAPKAP1, TPP2, TPR, PPP1R14C, AKT3, ELP2, RABL6, PROSC, CAMSAP3, UBE2H, TBCEL, ECT2, RTTN, DAPK1, UBE2N, LAP3, AMH, LARP6, XPC, UBE2K, BTG1, KRIT1, COMMD3, BUB1B, UBE2W, PRKCZ, PPP4R2, USP9X, NANOS1, KIAA0101, ZNF706, DDTL, TYMS, TUBGCP3, MOAP1, TUBGCP5, NPAS3, SPRR2D, POU2F3, ENO3, ERCC2, TXNIP, ICA1, DNM1L, MAP1B, RUFY1, KIF18A, MTL5, CENPE, CENPI, MON2, UBL5, SLC17A5, CEP68, SP4, ANXA11, BRE, CENPV, ACTR10 | 462 | 1.4 | 1.67E-15 |
| GO CC | GO:0005634~nucleus | TUBB2A, RPL15, NAA15, NAA16, CLK1, SART3, CDCA7, HIST1H2BN, CLK3, RAE1, CLK4, PATZ1, OGT, TIGD7, CCNA2, CDCA4, LUC7L3, ZNF101, KRR1, MAGI2, RCOR3, ZNF644, TIGD1, PIK3CB, ZNF48, LIG3, ZHX2, ZNF502, ESPL1, GEM, MECOM, ERGIC2, PTRF, MAPK6, JUN, TRAPPC2, CRTC2, ZNF611, ZNF79, ZSCAN5A, NTAN1, UBA5, PUS7, ADAP1, HESX1, ZNF226, RAC1, RCHY1, TCF3, NAT14, UNC45A, MKI67, PIBF1, NDC80, GCFC2, UPF3A, RERG, ZNF215, SRSF5, WDR61, PPP2R3C, SRSF1, ELF1, ZNF532, U2SURP, NAP1L1, ZNF347, CCNE1, HSF2, ANP32A, MASTL, BRD4, USP15, ATF7IP, MICAL2, ZSWIM7, ZNF138, FMN1, ZNF134, FANCD2, RRM2, GNB5, TXK, ZNF33B, CLOCK, ARL4A, SRGAP2, ZNF557, HIST1H2AE, POLA1, ERI1, NUFIP1, ZNF367, ZNF655, MYCBP2, MTMR2, ZNF169, SF3B1, SFR1, TCEA1, ZNF750, PER3, FBXW11, FEN1, TRIP13, ZNF267, ZNF263, SPATA33, ZNF566, HIST1H2BD, ZNF28, NUB1, NUCKS1, SWAP70, ZNF771, HIST1H2BG, CS, ZNF770, PHF10, CAPN2, APPL2, ZNF165, ATRX, DUSP3, RPL22, GSK3B, SFPQ, SYF2, ZNF461, MPHOSPH6, MPHOSPH8, AKNA, SRP14, ZNF580, EIF5, RBM4, STYX, ZNF451, TCEAL1, TCEAL4, CDKN2C, DNAJC9, ZNF302, CTDSP1, ASPM, MTUS1, EGFR, BCL10, RBFOX2, DFFB, NEIL3, RELB, TOPBP1, GRHL2, RMI1, PKIA, TACC1, NABP1, DCUN1D1, HIF1A, SYCP3, PRCC, HSPB8, ZNF586, HMGB1, EID1, CNBP, MPLKIP, BBS7, SLF2, SLF1, DSCR3, SESN2, ATF1, EPM2AIP1, TCERG1, PTK6, HIST1H4E, RPL4, HELLS, GINS1, FKTN, FOXA1, BRIP1, WHSC1, SPRYD4, CDKN1A, ATF3, EAF1, ZNF317, PNRC2, DNAJB2, RNPC3, HIST1H3E, NBN, GPBP1, ALG2, DICER1, E2F8, SLFN5, FER, MBP, PRMT7, LRWD1, CASP2, KDM5B, NFX1, KIF14, CDK1, CCNL1, NUSAP1, IRF2BP2, HMGA2, CHAMP1, CDK2, TMEM38B, TRNAU1AP, FAM120A, NSL1, TXNRD1, EMC2, UGP2, SNX10, KDM6B, SLC7A6OS, HYLS1, GDAP1, DCK, NR3C1, TSPYL4, TSC22D1, CHD9, TFAM, CHD2, VPS36, NSUN2, HSPA8, EXO1, NCDN, NF1, RCAN1, ANXA5, SMC2, SMC3, SMC4, INVS, ZBED4, ZRANB2, JAK2, ZRANB3, ALKBH3, SCAND1, ITGB3BP, RAD51C, PDLIM7, SLC35A2, ATP2B1, SFSWAP, N4BP2L2, H2AFV, WWP2, CREB3L2, H2AFX, FAM103A1, PAN2, YY1, KRT10, POLB, OPTN, ESCO2, TBRG1, CDCA7L, SRSF10, SRSF11, CHEK1, IGF2BP2, AFAP1L1, PXK, VRK2, HOXA10, FBXO5, TCF25, IKZF5, PELI1, TKT, SMYD2, NOP10, RAD54L, NOTCH2, RNF7, GRK5, NCOR1, CPEB2, EID2B, FERMT2, SOBP, SHOC2, SENP5, ARL2BP, CAMKK2, PARN, BLZF1, ANKRD11, NSMCE2, TOP2A, IP6K2, CTBP2, DSN1, UFD1L, HERC6, SF1, MBD4, FAM76B, FAM76A, RAD52, MBD1, ZCCHC17, GADD45B, GADD45A, MECR, USP3, POLR2K, LARP1B, MLF1, SUMO3, ZFP36L1, NCAPG, THAP1, ACTL6A, RAB2A, UBXN1, PDS5B, BIRC5, HNRNPDL, PAPOLA, SLC50A1, KIF20B, APBB2, CALM1, ZC3HAV1, EIF5B, SAE1, PMAIP1, MCM10, CBX5, SMNDC1, TOP1, ZNF181, GSTM3, ORC5, LOX, ATOH7, AKIRIN1, CUTC, RBMS1, DTL, USP1, TOR1AIP1, RRP8, NUPL2, STK4, ZNF197, FGFR1OP, MCMBP, SLU7, AKAP7, DST, MAP7D3, CAV2, BLM, SOX4, SOX6, CMPK1, CSE1L, MTCH2, C12ORF10, MFAP3L, STRBP, SSX2IP, SLC30A9, RNF14, MAFG, PARD6B, CSTF3, CSTF2, KCTD1, ATAD2, CASC5, HEATR1, BRCA1, PRICKLE4, UACA, PSMC4, WDR4, CPNE3, TMPO, SMC1A, PDCD6, DUT, C9ORF72, ARHGAP19, HIST2H4A, PDCD2, NFATC2IP, PHC3, FUBP1, RANBP9, MAPKAP1, TPP2, SETMAR, CDK12, TPR, RABL6, ZNF92, ECT2, UBE2N, LAP3, LARP6, XPC, UBE2K, BTG1, COMMD3, UBE2W, MSANTD4, CUX1, SUPT3H, PPP4R2, KIAA0101, ZNF706, TYMS, ERCC8, NPAS2, POU2F3, ERCC2, TXNIP, DLST, KAT2B, RAD51AP1, RUFY1, KIF18A, MTL5, CENPE, CENPI, RALGDS, UBL5, ADNP2, CEP68, SP4, BRE, CENPV, CENPU, NFIC, NFIA, ZBTB8A | 454 | 1.31 | 1.49E-09 |
| GO CC | GO:0005829~cytosol | ITGB3BP, MOCOS, EHHADH, RPL15, LPAR1, TPK1, CUL3, COL4A3BP, PIK3CA, DEPDC1B, OGT, EIF2B2, PAN2, PAN3, PIK3CB, MYH3, NUDT15, ESPL1, VPS41, OPTN, MECOM, FARP1, RPTOR, BCL2L11, TANK, PGM2, PGM3, PTRF, MAPK6, JUN, PUDP, MAPK9, NUP43, TRAPPC2, PFKFB4, UBA5, IGF2BP2, BCL2L2, CHEK1, BCL2L1, PPCDC, RIC1, EPHB2, TK1, GPD1L, NPHP3, RILPL1, STX17, RAC1, CDA, FBXO5, TNKS, AMD1, PELI1, OSBPL3, TGFBR2, GARS, ACACA, TRIO, CDC23, GAS2, NDC80, TKT, SMYD2, GART, NOTCH3, UPF3A, RERG, RPE, CCT8, SH3RF1, ENOX2, UBE2G2, FERMT2, LATS1, ARL2BP, CCNE2, CCNE1, PARN, AASDHPPT, FXN, EEF2K, ARHGAP11A, RAP2A, PRTFDC1, RAP2C, DSN1, POLR1D, UFD1L, RAB4A, HERC6, CCT6A, C8ORF44-SGK3, ARHGEF10, CHMP1B, PAPD4, MB21D1, SERPINB8, RRM2, EIF4A2, PSEN2, GNB5, THEM4, PARVA, SRGAP2, SERP1, BID, ATG10, LIMS1, SORD, EXOC7, EXOC8, POLR2K, PPFIA1, RPL27A, UROS, RPL37, FKBP1A, ZFP36L1, MTMR2, RPL32, KLC1, SQSTM1, NCAPG, CHM, FBXW11, MLLT4, PLEC, CSNK1A1, UBXN1, SPATA33, SLC8A1, PDS5B, SWAP70, BIRC5, CAPN2, RPL28, MSRB3, RPS6KA3, DUSP3, PSMD12, RPL22, PYGL, GSK3B, GFPT1, NLN, PAICS, CALM1, SRP14, CEP72, STIL, OCLN, AP1G1, EIF5, CEP78, F2RL1, STYX, EIF5B, SAE1, PMAIP1, PI4K2B, VCL, GSTM3, ATAT1, CDKN2C, SLC2A1, MLKL, BPNT1, BCL10, CEP89, DFFB, RELB, CORO7, FADD, NUPL2, CDO1, STK4, TNNT3, HIF1A, FGFR1OP, NBR1, USO1, AKAP7, STMN1, DST, CHMP2A, ANAPC16, HMGB1, CNBP, ARFGAP3, BBS7, DIAPH2, STAM2, SESN2, CMPK1, RASAL2, BLOC1S4, AKT1S1, CSE1L, PRKRA, RPL4, SEC61A1, TRAF3, ARHGDIB, ERCC6L, PARD6B, GABARAPL2, CFLAR, RPGRIP1L, FN3KRP, CASC5, MYL12A, VAV2, ATG3, WIPI1, KCTD7, CDKN1A, PLK4, PSMC4, GLS, PRKAR1A, DNAJB2, CPNE3, HSPD1, FPGS, SMC1A, NBN, PRKAG2, DICER1, ARHGAP19, PPIP5K2, RHOQ, FER, MKLN1, RANBP9, PRMT7, TPP2, MAPKAP1, CASP8, RHOD, CASP2, SEC24D, KIF14, CDK1, KIF11, KIF15, ECT2, CDK2, UBE2N, EIF4G2, ATP6V1A, EIF4G3, NSL1, NUCB2, BUB1B, TXNRD1, INPP4B, CUX1, SMS, UGP2, PRKCZ, USP9X, CALD1, ADH5, DCK, RPS15A, NR3C1, TPM4, FAM13B, TYMS, TUBGCP3, TFAM, NPAS2, MOAP1, TUBGCP5, ENO3, BRK1, TNRC6B, VPS36, TNRC6A, HBB, HSPA8, CENPO, TXNIP, SCLT1, CENPN, ICA1, DNM1L, KAT2B, NCDN, NF1, MAP1B, KIF18A, AHI1, CENPE, TPMT, ANXA5, SMC2, SMC3, CDC25A, RALGDS, CENPI, SMC4, CADPS, MPI, GSPT1, AKR1B1, JAK2, CENPU, SH3D19, CUL4B | 311 | 1.41 | 3.15E-09 |
| GO CC | GO:0005654~nucleoplasm | ITGB3BP, RAD51C, FAM20B, PNISR, ZNF638, SART3, CUL3, HIST1H2BN, CDCA7, CLK3, COL4A3BP, CREB3L2, H2AFX, PATZ1, OGT, CCNA2, LUC7L3, LIG3, ZHX2, POLB, OPTN, MECOM, RPTOR, ESCO2, RFC5, DCAF7, PTRF, CEP350, MAPK6, JUN, MAPK9, SCAF8, COASY, CRTC2, SRSF10, SRSF11, CHEK1, RILPL1, FBXO5, RCHY1, TCF3, KLF5, GARS, SMYD3, CDC23, TKT, SMYD2, RAD54L, GCFC2, FNIP2, NOTCH3, UPF3A, SRSF3, NOTCH2, SRSF5, RNF7, WDR61, PARPBP, CCT8, NCOR1, CREBRF, SRSF1, CLSPN, COPS2, ELF1, FERMT2, U2SURP, VPS37A, SHOC2, TRMT10C, SENP5, BOD1L1, CCNE2, CCNE1, BLZF1, FANCI, ANKRD11, ANP32A, NSMCE2, MASTL, BRD4, TOP2A, DEDD2, IP6K2, ATF7IP, UFD1L, POLR1D, SF1, MBD4, RAD52, RBBP6, FANCD2, RRM2, ZNF480, RBM39, GADD45A, NSD1, CLOCK, ARL4A, CLUAP1, POLR2K, POLA1, ZNF367, FAAP20, SUMO3, SF3B1, SRRT, MRPL10, STK40, SQSTM1, TCEA1, ACTL6A, SNAP23, MLLT4, FEN1, RBM25, ZNF263, ECI2, TRMU, HIST1H2BD, PDS5B, PPHLN1, HIST1H2BG, BIRC5, HNRNPDL, COG3, RPS6KA3, DUSP3, PAPOLA, PSMD12, SFPQ, KIF20B, USP48, RBM15, CALM1, RBM4, SAE1, MCM10, TCEAL1, PNN, CBX5, TOP1, KLHL8, ORC5, CTDSP1, AKIRIN1, NQO2, ORC3, ARGLU1, RBFOX2, DTL, DFFB, USP1, NEIL3, RELB, TOPBP1, RRP8, NUPL2, GRHL2, RMI1, ELL2, PTHLH, NABP1, HIF1A, HSPB8, MCMBP, SLU7, MAP7D3, ANAPC16, EID1, HMGB1, MPLKIP, EID3, BLM, STAM2, SOX4, SOX6, NR2C2, ATF1, AKT1S1, CSE1L, POLE2, C12ORF10, PTK6, PRKRA, HIST1H4E, ERCC6L, GINS1, MAFG, CSTF3, CSTF2, BRIP1, ATAD2, CASC5, WHSC1, BRCA1, SAFB2, TRNT1, CDKN1A, ATF3, EAF1, PSMC4, SMARCC1, WDR4, C2ORF49, FBXO32, ZNF318, HIST1H3E, RNPC3, SMC1A, DUT, C9ORF72, NBN, E2F3, GPBP1, PRKAG2, HIST2H4A, FAM63B, PHC3, FUBP1, PRMT7, MAPKAP1, CASP8, TPR, KDM5B, AKT3, CDK1, BANP, HMGA2, CHAMP1, ZFR, CDK2, UBE2N, LAP3, XPC, CUX1, KDM6B, MATR3, HYPK, SMARCAD1, SUPT3H, PPP4R2, HAUS6, KIAA0101, NR3C1, TYMS, CHD9, ERCC8, NPAS2, NPAS3, POU2F3, CHD2, NSUN2, TNRC6A, HSPA8, ERCC2, PPP4R3B, EXO1, CENPO, CENPN, KAT2B, RAD51AP1, MSH2, FAM188A, SMC2, SMC3, CDC25A, CENPI, SMC4, SP4, AKR1B1, ANXA11, BRE, ZRANB2, CENPV, JAK2, CUL4B, MIS18BP1, SH3D19, CENPU, SETD2, ALKBH3, NFIA | 283 | 1.61 | 2.66E-15 |
| GO CC | GO:0016020~membrane | ITGB3BP, AKNA, IMPAD1, AP1G1, HBS1L, RPL15, NAA15, STRN, PI4K2B, MTHFD1L, PNN, ATP2B1, CUL3, CISD2, SLC16A1, CLK3, WWP2, SLC2A1, LRRC59, RNF149, EGFR, KRR1, SLC33A1, RPS6KC1, KRT10, CORO7, VPS41, CHPT1, ERGIC1, GCC2, TACC1, ERGIC2, PARP16, CEP350, LRP10, ATP2C1, NBR1, ACAP2, USO1, SLU7, RIPK4, STMN1, ARL8B, MAP7D3, CHMP2A, CAV2, ARFGAP3, BBS7, PNPT1, SNX4, PRRC2C, RIC1, IKBIP, MIA3, KIAA2013, CSE1L, MTCH2, RAC1, PRKRA, HIST1H4E, HLA-DPB1, RPL4, ARL6IP5, SEC61A1, ERCC6L, ARHGDIB, OSBPL3, MKI67, TGFBR1, NDC80, HEATR1, GAS2, PPIF, RERG, APOL2, NOTCH2, DDX55, ATP2A2, PSMC4, PRKAR1A, HIST1H3E, HSPD1, TMPO, ALG11, NCOR1, CYB5R3, SLC20A1, ALG2, ATL3, KIAA0430, NAP1L1, TMEM237, DNAJC10, NUP188, HIST2H4A, SLC26A2, MMP25, PIGK, SRPX, FANCI, DENND5B, FAM129A, PPP1R14C, CASP2, PIGA, AP3B1, KIF14, CDK1, KIF11, MAN1A2, KIF15, PIGT, PIGN, EIF4G2, FAM120A, PSEN2, AVEN, SEC23B, MATR3, BID, MFSD6, PRKCZ, SORD, EXOC7, GALNT7, EXOC8, USP9X, GDAP1, RPL27A, RPS15A, FKBP1A, NR3C1, ESYT1, TPM4, MYCBP2, NDC1, TUBGCP3, DGKE, RPL32, NCAPG, KLC1, BCAP29, PCSK6, ACSL4, INPP5A, HSPA8, FEN1, CSNK1A1, DLST, ECI2, DNM1L, NCDN, MSH2, NF1, CENPE, ANXA5, RPL28, ANXA3, ITPR2, SLC17A5, INVS, PSMD12, MBOAT7, ANXA11, TENM3, DYM, DPM3, SYNM, APBB2, PAICS | 170 | 1.3 | 0.0178 |
| GO CC | GO:0005813~centrosome | STIL, CEP72, CEP57L1, CEP78, VPS37A, ARL2BP, HOOK3, CCNE1, SLC16A1, CCSAP, MASTL, CEP112, CDK1, CEP89, DTL, KIF15, ESPL1, TOPBP1, KIAA0586, CAMSAP3, ARHGEF10, CDK2, RTTN, ELL2, ANKRD26, CEP350, FGFR1OP, PSEN2, SNX10, MAP7D3, SNAP29, CLUAP1, PPP4R2, HAUS6, MPLKIP, BBS7, HYLS1, CEP126, SLF1, CHEK1, BCL2L1, TUBGCP3, RILPL1, TUBGCP5, NCAPG, FBXW11, TBC1D31, ERCC6L, CSNK1A1, PPP4R3B, SCLT1, NIN, RPGRIP1L, AHI1, PIBF1, PLK4, CEP68, GSK3B, SLAIN2, CCT8, KIF20B, PPP2R3C, CALM1 | 63 | 2.21 | 6.99E-07 |
| GO CC | GO:0005694~chromosome | PDS5B, DTL, PPHLN1, NUSAP1, TOPBP1, WHSC1, CENPE, RBBP6, FAAP20, ZFR, SMC3, BRCA1, BOD1L1, BRD4, SETD2, SMC1A, NSD1, CLOCK | 18 | 3.16 | 4.40E-03 |
