# Supplementary material for: Silencing of miR-182 is associated with modulation of tumorigenesis through apoptosis induction in an experimental model of colorectal cancer
Source: BMC Cancer. 2019 Aug 20;19:821. doi: 10.1186/s12885-019-5982-9 (PMC6700772; doi:10.1186/s12885-019-5982-9)
Supplement: Supplementary file 4 — Table S4. MiR-182 predicted target transcripts for which differentially expression in MICOL-14h-tert and/or MICOL-14tum cells after treatment was confirmed by RT-PCR. The table showed the transcripts and the correspondinggenes, probesets and Taqman Assay ID used for experimental qRT-PCR validation. For each probeset and cell line, the expression variation observed according to Primeview Microarray data analysis is shown as LogFC of the anti-miR-182 vs anti-miR-NC comparison; values corresponding to a stastistically significant differential expression are in bold. (DOCX 19 kb) [file 12885_2019_5982_MOESM4_ESM.docx]

**Supplementary Table 4. MiR-182 predicted target transcripts for which differentially expression in MICOL-14^h-tert^ and/or MICOL-14^tum^ cells after treatment was confirmed by RT-PCR.** The table showed the transcripts and the correspondinggenes, probesets and Taqman Assay ID used for experimental qRT-PCR validation. For each probeset and cell line, the expression variation observed according to Primeview Microarray data analysis is shown as LogFC of the anti-miR-182 *vs* anti-miR-NC comparison; values corresponding to a stastistically significant differential expression are in bold.

| **TARGET GENE** | **TRANSCRIPTS** | **ASSAY ID** | **Probeset ID** | **LogFC MICOL14h-tert** | **LogFC MICOL14tum** |
| --- | --- | --- | --- | --- | --- |
|  |  |  |  |  |  |
| HIST1H2BH | ENST00000619466.2 | Hs00374322_s1 | 11759111_x_at | **1.06** | **0.65** |
| NABP1 | ENST00000425611.6 ENST00000410026.6 | Hs00224567_m1 | 11726725_a_at | **0.75** | **0.81** |
|  |  |  | 11726726_a_at | **0.71** | **0.76** |
|  |  |  | 11726727_a_at | **0.75** | **0.72** |
|  | ENST00000425611.6 | Hs00224567_m1 | 11726728_a_at | **0.48** | **0.5** |
| RND3 | ENST00000375734.6 ENST00000263895.8 | Hs01003594_m1 | 11753427_a_at | **0.35** | **0.43** |
| TRIO | ENST00000344204.8 ENST00000513206.5 | Hs01125865_m1 | 11724261_a_at | **0.74** | **1.3** |
|  |  |  | 11744590_a_at | **0.14** | 0.4 |
